# Optimal electric vehicle charging stations and distributed generation placement by partitioning the distribution network using the modified newman fast algorithm

**DOI:** 10.1038/s41598-026-35433-5

**Published:** 2026-02-11

**Authors:** Mohamed Ahmed Ebrahim Mohamed, Asmaa Nasser Abdellatif Gawish, Mohamed Eladly Metwally

**Affiliations:** 1 Electrical Engineering Department, Faculty of Engineering at Shoubra, Benha University, Cairo, Egypt; 2 Department of Electrical Engineering, El Shorouk High Institute of Engineering, Cairo, Egypt

**Keywords:** Community detection, Distributed generator, Electric vehicle-charging station, Electrical coupling strength, Electrical modularity, Modified newman fast algorithm, Energy science and technology, Engineering, Mathematics and computing

## Abstract

This article introduces a novel and numerically validated framework for the well-optimized placement and capacity selection of Distributed Generation (DG) units and Electric Vehicle Charging Stations (EV-CSs) in power distribution networks (PDNs). The methodology employs a Modified Newman Fast Algorithm (NFA) enhanced with Electrical Coupling Strength (ECS) to partition the network into electrically cohesive Virtual Microgrids (VMs). Within each VM, resources are optimally allocated using two recent metaheuristic techniques: the Starfish Optimization (SFO) and the Puma Optimization (PO) methods and compared against the conventional Particle Swarm Optimization (PSO) approach. Each approach is executed for 500 iterations with 30 search agents. The discussed framework is tested on the IEEE 33-bus and IEEE 118-bus PDNs. For the 33-bus PDN, the approach minimized active power losses by approximately 82%, improved the lowest bus voltage magnitude from 0.8361 p.u to 0.979 p.u, and increased the Stability Index (SI) from 0.6256 p.u to 0.927 p.u. For the 118-bus network, real-power losses were decreased by 68–69%, with notable enhancements in both voltage profile and SI. Additionally, PO demonstrated the fastest convergence speed among the tested algorithms, confirming its suitability for large-scale optimization. The study results demonstrate the effectiveness of the presented VM-based co-allocation strategy in enhancing power system performance and scalability, with future work focusing on cost-aware multi-objective optimization and real-world deployment in Egyptian PDNs.

## Introduction

The universal demand for fossil fuels continues to grow significantly, particularly in the electricity generation and transportation sectors. However, conventional energy sources are not only expensive but also major contributors to air pollution and CO₂ emissions. According to prior studies, the transportation sector is expected to grow by 54% by 2035, leading to substantial increases in environmental pollution and energy-related costs^1–3^. In response, many countries are shifting from diesel engine vehicles to sustainable alternatives such as electric vehicles (EVs), owing to their environmental and economic advantages^4^. EVs produce lower CO₂ emissions in contrast to fossil fuel-powered vehicles, even when accounting for the full lifecycle of energy production, making them a promising solution to future transportation and environmental challenges. However, incorporating Electric Vehicle Charging Stations (EV-CSs) into power distribution networks (PDNs) presents considerable operational challenges^4–6^. These include increased energy demand and line loading, leading to elevated power losses, voltage deviations, and potential instability, ultimately compromising system reliability, efficiency, and loadability^7^. A critical challenge lies in detecting the best possible place and capacity of EV-CSs during the planning and operational phases. To mitigate these challenges, coordinated allocation of Distributed Generators (DGs) units such as synchronous generators, photovoltaic (PV) modules, wind turbines, and capacitor banks alongside EV-CSs has been proposed. DGs can relieve line congestion, support local voltage levels, and improve overall power quality^8–10^. In this study, the Zebra Optimization (ZO) approach is employed to detect the best size and location of various models of DGs, and its performance is benchmarked against existing optimization methods. Furthermore, the study investigates the impact of deploying a single DG versus multiple DG units within the network^11–13^. Recent works such as^14^ and^15^ have proposed improved swarm intelligence and bio-inspired metaheuristics for distribution network optimization, demonstrating strong convergence properties and robustness under complex constraints. Several studies have suggested that co-optimizing the placement of EV-CSs and DGs results in superior technical and economic benefits compared to treating them separately^10,16–18^. Other works emphasize geospatial allocation of EV-CSs, using geographic test system maps to demonstrate practical feasibility. For instance, researchers have incorporated EV-CSs and PV-type DGs applying the Archimedes Optimization (AO) method to minimize power losses by optimally placing charging stations and PV units^11,19^. Results are often compared with results from other metaheuristic algorithms, such as the Cuckoo Search (CS) approach, Particle Swarm Optimization (PSO), and others. Further optimization efforts using Genetic Algorithms (GA) and Bee Colony (BC) approaches have been applied to the joint placing and selecting capacity of EV-CSs and DGs^20,21^. In the context of resource and EV-CS placement, the voltage controller application offers clear environmental merits. By controlling absolute voltages within allowable bounds, the voltage controller decreases active losses across the PDN^22^. Reducing losses translates into decreasing the generation demands, which in turn minimizes fuel utilization and CO2 emissions in networks still depending on dispatched power plants. Moreover, efficient voltage regulation enhances the ability of the system to accommodate renewable resources, thereby enabling a higher implementation of clean sources such as solar PV. This synergy between DG integration and voltage control contributes beneficially to Sustainable purpose^23^. Nevertheless, potential environmental demerits should also be considered. Frequent procedure of the equipment of voltage control (e.g, on-load tap changers, reactive power compensators, or inverter-based controllers) may lead to increased equipment wear, shortening their service life and requiring more frequent replacements. The manufacturing and deployment of advanced electronic controllers also involve embodied energy and material usage, contributing indirectly to environmental impacts^24,25^. This study^26^ investigates the optimal sizing and placement of EV-CS integrated with DG in a metropolitan PDN using a hybrid GA-modified slap swarm algorithm (HGA-MSSA). Simulation results across three EV-CS and DG integration scenarios show significant improvements in network utilization (up to 79% in G2V mode). Similarity in^27^,the optimum allocation of EV-CSs, shunt capacitors, and DGs in reconfigured radial PDN using a fuzzified multi-objective function. An improved hummingbird algorithm (COLAHA) is proposed, demonstrate superior convergence and performance relative to other metaheuristic algorithms. To handle the increasing complexity of modern PDNs, the concept of Virtual Microgrids (VMs) has gained prominence, wherein networks are partitioned into smaller, loosely coupled regions to simplify optimization and enable decentralized control^28–30^. Recent research proposes modular and distributed resource allocation frameworks within VMs^31–34^, incorporating tools from graph theory and complex network analysis^35^. For example, in^36^, a two-stage optimization framework uses probabilistic EV demand modeling, hierarchical clustering, and the Galaxy Gravity (GG) method to reduce energy losses and voltage deviations while enhancing system reliability under both electrical and transportation constraints. Similarly^37^, utilizes K-means clustering with Loss Sensitivity (LS) factors and voltage deviation minimization to strategically site single-unit and multiple units of resources. A hybrid approach combining complex network theory and multi-objective Genetic Algorithms is proposed in^33^, aiming to transform conventional PDNs into interconnected VMs. Additionally^38^, presents a three-layer active planning model for the PDN, incorporating a two-level VM-based optimization method that leverages structural network features and hierarchical decision-making for DG allocation. While the Newman Fast Algorithm (NFA) has been extensively applied in community selection problems^39,40^, its use in power systems has been limited due to its inability to handle hierarchical network structures or account for electrical distances. In addition, several techniques exist for dividing PDN into VMs. Impedance-based partitioning captures the electrical distance between buses, but it neglects branch capacity and its thermal limits. As a result, it may group nodes that are topologically close yet weakly coupled in practice. In contrast, the Electrical Coupling Strength (ECS) approach extends impedance-based metrics by incorporating line capacity and composite weight indices, thereby ensuring that clusters are both electrically close and operationally viable. Another method in^41,42^ is based on power flow sensitivity factors (e.g., PTDF), which reflect the actual impact of power injections online flows. While insightful, these metrics are highly dependent on the current operating point, meaning that partitions may change significantly with variations in load or generation. ECS, by comparison, is less sensitive to operating conditions and thus provides more stable partitions across different scenarios. Therefore, ECS itself combines electrical distance, line capacity, and equivalent weight indices to produce VM that are electrically cohesive and resilient. By balancing structural proximity with operational feasibility, ECS yields self-sufficient and physically meaningful communities. For this reason, ECS was chosen in this study, as it preserves electrical cohesiveness better than impedance-only methods, which are too simplistic, or flow-sensitivity metrics, which tend to be unstable. To address these limitations, a Modified NFA is proposed in^28^, replacing topological adjacency with ECS^30,40,43,44^, which enables the detection of functional communities with strong internal electrical cohesion, ideal for decentralized control and planning. Although this methodology is primarily applied to high-voltage transmission systems^40^, it shows strong potential for extension to medium and low voltage PDN and EV-CS allocation, which remains an open research challenge. To fill this gap, the presented article introduces a novel clustering-based framework for partitioning PDNs into Virtual Microgrids, aiming to co-optimize the allocation of DGs and EV-CSs. The DG type employed in this paper is a synchronous generator, as used in prior works^8,11,12,45,46^. The primary optimization purposes are to reduce total power losses and raise the stability index (SI), which serves as the key objective function.

### Primary contributions

The major participants of this research are outlined below:

1. Enhancement of a modified NFA for clustering electrical PDNs into VMs. The proposed method replaces conventional topological adjacency with ECS, enabling the identification of electrically cohesive communities that better reflect real power flow interactions and interdependencies among network nodes.
2. Implementation of two recent metaheuristic optimization techniques, namely the Starfish-Optimization (SFO)^47^ and the Puma Optimization (PO)^48^methods to co-allocate EV-CSs and DG units within the identified VMs.
3. Performance benchmarking of the proposed algorithms (SFO and PO) against the established Particle Swarm Optimization (PSO) approach^49^. The comparison is considering technical performance metrics, including total loss of real power reduction, enhancement of the SI, maximum Loadability index (LLI%), maximum voltage deviation (VD), and maximum hosting capacity of EV, demonstrating the importance of the suggested approach in clustered PDNs, as well as the convergence curve of each algorithm.
4. Applying the Wilcoxon statistical test to ensure robustness and resilience of the proposed techniques.
5. Compare the results of this research with other research papers.
6. A real-time model was also added in the last section, considering the uncoordinated charging of EVs with providing DGs (wind turbine-based), considering its uncertainties, so it will support PDN efficiency.

### Paper outline

The other sectors of this study are overviewed as follows: Section “Partitioning methodology” illustrates the recommended strategy, which involves clustering the PDN into VMs using the Modified NFA based on ECS. Section “Allocate electric vehicle charging stations and resources” describes the optimal allocation strategy for EV-CSs and DGs within each VM using the SFO, PO, and PSO for comparison with their two tested systems. Section “Adding wind energy resources in each Virtual microgrid” illustrates the conversion of the system to a real-time system. Section “Results and discussion” proposes the study models and simulation results on benchmark systems, highlighting the effectiveness and reliability of the discussed strategy also the stochastic behavior of EV with allocation wind turbine-based DG was discussed and its impact. Finally, Section “Conclusion” briefs the paperwork and highlights future research directions.

## Partitioning methodology

### Electrical coupling strength

The problem formulation begins by partitioning the PDN into VMs, considering the ECS-matrix^28^ to The ECS is a function of the effective impedance ${Z}_{ij}^{E}$ and the power capacity of each line ${C}_{ij}$. These parameters are calculated using Equations (1) and (2), respectively, and serve as the foundation for quantifying the electrical interaction strength between network nodes.

1 $$Z_{ij}^{E} = {\kern 1pt} \,Z_{ij} \, + \,Z_{jj} - 2Z_{ij} \quad i,j \in N_{B}$$

Where ${Z}_{ii}$, ${Z}_{jj}$,and ${Z}_{ij}$ are the impedance matrix elements of the PDN, and ${N}_{B}$ is the system bus number.

2 $${C}_{ij}=\mathit{min}\left(\frac{{P}_{\mathit{max}l}}{\left|{PTDF}_{ij}^{l}\right|}\right) i,j\in {N}_{B}, l\in {N}_{L}$$

${P}_{\mathit{max}l}$ is the power flow line limit for the branch $l$, ${N}_{L}$ is the network line number. ${PTDF}_{ij}^{l}$ is the power transmitted distribution factor based on the line power change $l$ when 1 p.u. power is supplied to bus $i$ drawn from bus $j$^44,50–52^.

3 $${ECS}_{ij}=\left|\alpha {\overline{Y} }_{ij}+j\beta {\overline{C} }_{ij}\right|$$

4 $${\overline{Y} }_{ij}=\frac{{Y}_{ij}}{\overline{Y} }$$

5 $${\overline{\mathrm{C}} }_{\mathrm{ij}}=\frac{{\mathrm{C}}_{\mathrm{ij}}}{\overline{\mathrm{C}} }$$

6 $${\mathrm{Y}}_{\mathrm{ij}}=\frac{1}{{\mathrm{Z}}_{\mathrm{ij}}^{\mathrm{E}}}$$

The primary objective of a PDN is to deliver maximum electrical power with minimal losses. Therefore, shorter electrical distances (i.e., lower effective impedance) and higher transmission capacities typically indicate a stronger electrical coupling between two buses. Accordingly, the ECS between buses is defined as in^38^.

where *α* and *β* are proportion coefficients, are both assumed to equal 0.5 $\left(\alpha +\beta =1 \right)$ and $\overline{C }$ the average transmitted power capacity, and ${Y}_{ij}$ is the element of effective admittance matrix. $\overline{Y }$ is the average equivalent admittance.

The effective impedance and capacity of the transmission line are two tunable parameters that influence the impact of each line on the ECS. By incorporating ECS, the conventional topological adjacency matrix is transformed into an ECS-based power network matrix, as expressed in Equation (3).

### Electrical modularity ${Q}_{e}$

The community detection process is carried out using the Modified NFA, which operates based on the ECS matrix. In each iteration, the algorithm evaluates the electrical modularity ${Q}_{e}$ to detect the optimal partitioning of PDN^29^. The electrical modularity quantifies the quality of the detected communities by considering both electrical and structural characteristics of the network. A well-partitioned VM structure is characterized by a higher density of internal connections compared to those in a randomly partitioned network. Consequently, a higher value of ${Q}_{e}$ indicates a more effective and meaningful partitioning of the PDN into VMs.

7 $${Q}_{e}=\frac{1}{2m}\sum_{ij}^{NB}\left[{ECS}_{ij}-{ECS}_{i}{ECS}_{j}\right] .\delta \left({c}_{i},{c}_{j}\right) i,j\in {N}_{B}$$

8 $$m=\frac{1}{2}\sum_{ij}^{NB}{ECS}_{ij} i,j\in {N}_{B}$$

where $m$ is the total ECS of the network, which ${ECS}_{ij}={ECS}_{ji}$.

9 $${ECS}_{i}=\sum_{v}^{NB}{ECS}_{iv} i,j\in {N}_{B}$$

${ECS}_{i}$ is the degree of the ECS matrix at the bus $i$, so it is the sum of the row or column $i$ in the ECS matrix. where ${c}_{i}$ and ${c}_{j}$ is the number of communities of bus $i$ and $j$ respectively. Additionally, Kronecker delta $\text{is }\delta \left({c}_{i},{c}_{j}\right)$, it’s equal to one if the node $i$ and $j$ belong to the same VM; otherwise, it’s equal to zero.

10 $${\Delta Q}_{{e}_{ n+1}}={Q}_{{e}_{n+1}}-{Q}_{{e}_{n}} n\in {N}_{B}$$

${\Delta Q}_{{e}_{ n+1}}$ is the change in electrical modularity in the current step.

###  Modified newman fast algorithm using electrical coupling strength matrix^40^

- Initialization: start with each bus being its own cluster. Then, calculate the initial electrical modularity ${Q}_{e}$.
- Check Connections: Use the original adjacency matrix to detect direct connections between VMs, merging only those that are directly connected.
- Merge by electrical Modularity Gain: calculate the modularity gain $\Delta {Q}_{e}$ for all valid merges using ECS weights. Then, combine the pair with the highest incremental.
- Repeat Merging**:** Repeat this step until all nodes are in one community (up to N−1 merges).
- Select Optimal Partition: evaluate ${Q}_{e}$ at each step to detect the best number of VMs for the PDN.

## Allocate electric vehicle charging stations and resources

The partitioning objective of PDN into zones the, referred to as VMs, is to enable the optimal allocation of EV-CSs within each localized region. Following the identification of electrically cohesive communities in the test system, DGs are allocated to lower energy losses and maximize the SI, as defined in Equation (11). To further enhance system performance, DG units are placed within each VM, specifically at the bus exhibiting the lowest SI value, thereby reinforcing voltage levels and ensuring they remain within acceptable operational limits. The optimization process is carried out using the SFO and the PO, and the results are benchmarked against those obtained using the conventional PSO method.

i. Multi-objective function
  11 $$F={w}_{1}{f}_{1}+{w}_{2}{f}_{2}$$
  12 $${f}_{1}=\mathrm{min}(T{P}_{loss} )$$
  13 $${f}_{2}=\mathrm{max}({SI}_{j})$$
  14 $$T{P}_{loss}=\sum_{lz=1}^{NL}{\left({I}_{l}-{I}_{DG}+{I}_{CS}\right)}^{2}\times {R}_{l}$$
  15 $${SI}_{j}={V}_{i}^{4}-4\times \left({P}_{j}{\times R}_{ij}+{Q}_{j}{\times X}_{ij}\right)\times {V}_{i}^{4}-4\times \left({P}_{j}{\times X}_{ij}-{Q}_{j}\times {R}_{ij}\right)$$

where:

**Table:** 

$F$	multi-objective function
${f}_{1}$	minimization of total active power losses
${f}_{2}$	maximization of the SI
${w}_{1}$ and ${w}_{2}$	are the weight factors, thus $({w}_{1}+{w}_{2}=1)$
$T{P}_{loss}$	total real power losses of the PDN
${SI}_{j}$	stability index of bus $j$
${I}_{l}$	line current
${I}_{DG}$	current injected from DG
${I}_{CS}$	current draw by CS
${R}_{l}$	line resistance of line $l$
${P}_{j}$	real power of $j$
${Q}_{j}$	imaginary power of $j$
${X}_{ij}$	reactance between $i$ and $j$
${V}_{i}$	absolute bus voltage of bus $i$

- ii- Constraintswhere:
  16 $$0.95{\le V}_{i}\le 1.05 p.u$$
  17 $${I}_{l}\le {I}_{l, Thermal}$$
  18 $$CCSq\_min \le CCSq \le CCSq\_max$$
  19 $$DGq\_min \le DGq \le DGq\_max$$
  20 $$\sum_{a=1}^{NG}{Q}_{Gen,a}=Total {Q}_{loss}+\sum_{c=1}^{ND}{Q}_{Load,c}$$
  21 $$\sum_{a=1}^{NG}{P}_{Gen,a}=Total {P}_{loss}+\sum_{c=1}^{ND}{P}_{Load,c}$$
  22 $$T{Q}_{loss}=\sum_{l=1}^{NL}{I}_{l}^{2}\times {X}_{l}$$

**Table:** 

${I}_{l, Thermal}$	thermal limit of each line
${P}_{Gen,a}$	active power generation by $a$ generator
${Q}_{Gen,a}$	reactive power generation by $a$ generator
${TQ}_{loss}$	total reactive power losses
${Q}_{Load,c}$	reactive power of load $c$
${P}_{Load,c}$	active power of load $c$

**Fig. 1:**
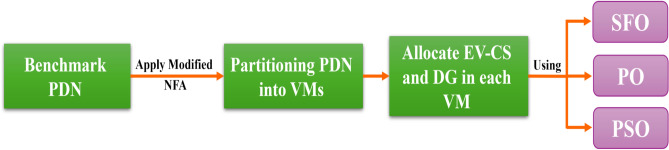
Overview of the proposed methodology.

To validate realistic integration of DG, the penetration level of DG is constrained so that the total DG size is below 60% of the total generation when the demand of EV-CSs is integrated^53^. Furthermore, the EV-CSs are modeled by utilizing Level-2 standard charger ratings, with each point of charging assumed to provide 3.3 kW, 6.6 kW, and 7.3 kW. Therefore, each CS contains approximately the total number of EVs dividing by the number of VMs. This is indicating how the EV-CS capacity was determined. Additionally, this reflects common deployment practices and ensures stable system operation under varying load conditions. It is worth noting that, as part of our ongoing research, we are extending this work to include detailed EV charging data from^54^. In that follow-up study, EV-CS charging points are considered, and DG sizing is dynamically updated based on the aggregated EV-CS demand, regulating the 60% penetration cap. This will lead to a more comprehensive analysis of sensitivity for EV-CS unit parameters and their effect on DG placement and PDN performance. The whole system procedure is shown in Figure 1.

### Optimization framework

#### Starfish optimization approach

To address complex engineering optimization problems, Changting Zhong developed a novel flock intelligence-based metaheuristic known as the SFO. This technique is inspired by the biological behaviors of starfish, which are mathematically modeled and implemented through a series of structured steps, as outlined below.

- Initialization

In this stage, the starfish’s position is generated randomly within its boundary of the obtained variable^47^

23 $$X={\left[\begin{array}{ccc}{X}_{11}& {X}_{12}& {X}_{1D}\\ \vdots & \vdots & \vdots \\ {X}_{N1}& {X}_{N2}& {X}_{ND}\end{array}\right]}_{N\times D}$$

where the starfish’s position matrix is X, whose size $N\times D$, where $N$ the number of populations, and D is the size of the obtained variable. the position of each starfish can be calculated as:

24 $${X}_{ij}={ l}_{j}+r \left({u}_{j}-{ l}_{j}\right), i=\mathrm{1,2} . . ., N, j=\mathrm{1,2},. . .,D$$

where r is a random value between 0 and 1, ${u}_{j}$ and ${l}_{j}$ are upper and lower boundaries with D size. Then, calculate the fitness value for each starfish

25 $$F={\left[\begin{array}{c}F\left({X}_{1}\right)\\ F\left({X}_{2}\right)\\ \begin{array}{c}\vdots \\ F\left({X}_{N}\right)\end{array}\end{array}\right]}_{N\times 1}$$

- Exploration

A hybrid search pattern is used to replicate starfish-seeking activities. To improve search efficiency, it uses a 5-dimensional pattern of search for higher dimensions, but a one-dimensional pattern for lower dimensions.

If $D>5$, then update the starfish position using Eq. (26) and Eq. (27)

26 $${Y}_{i,p}^{T}={X}_{i,p}^{T}+{a}_{1}\left({X}_{best,p}^{T}-{X}_{i,p}^{T}\right)\mathrm{cos}\theta , r\le 0.5$$

27 $${Y}_{i,p}^{T}={X}_{i,p}^{T}-{a}_{1}\left({X}_{best,p}^{T}-{X}_{i,p}^{T}\right)\mathit{sin}\theta , r>0.5$$

If $D\le 5$, calculate the updated position using Eq. (28)

28 $${Y}_{i,q}^{T}={E}_{t}{X}_{i,p}^{T}+{A}_{1}\left({X}_{k1,p}^{T}-{X}_{i,p}^{T}\right)+{A}_{2}\left({X}_{k2,p}^{T}-{X}_{i,p}^{T}\right)$$

${A}_{1}$ and ${A}_{2}$ are random values and ${E}_{t}$ is the starfish energy

- Exploitation

While the regeneration phase enables recovery from disadvantageous conditions, the intensification stage utilizes a two-directional search scheme during the predatory behavior.

i. Preying

The SFO utilizes a preying mechanism inspired by the natural food-gathering behavior of particles during the exploitation phase. This behavior is mathematically modeled as a parallel, bi-directional search method, which improves the algorithm’s capability to find the space of the solution efficiently. A critical step in this process involves computing the distance ${d}_{m}$ between the best-known (most promising) solution and the current positions of the remaining starfish in the population. Each individual then updates its position relative to this distance, guiding the search toward optimal solutions while preserving population diversity and avoiding premature convergence.

29 $${Y}_{i}^{T}={X}_{i}^{T}+{r}_{1}{d}_{m1}+{r}_{2}{d}_{m2}$$

${r}_{1}$ and ${r}_{2}$ represent random values. This permits starfish to improve their locations while retaining population diversity.

ii. Regeneration

The regeneration mechanism in the SFO is inspired by the biological ability of starfish to recover from injury through limb regrowth. This mechanism is crucial for enhancing global convergence and preventing this approach from getting stuck in local optima. In SFO, regeneration is selectively triggered for the lowest-ranked individual in the population, emulating the natural process wherein a starfish regenerates its lost limbs. By introducing new solution components into the population, this targeted regeneration step increases diversity and facilitates exploration of previously unvisited regions in the search space, thereby improving the algorithm’s robustness and search efficiency.

30 $${Y}_{i}^{T}={e}^{-\left(\frac{T.N}{{T}_{max}}\right).{ X}_{i}^{T}}$$

This rule enables the lowest-ranked starfish to gradually adjust its position over successive iterations, effectively simulating the biological process of regeneration.

31 $${X}_{i,p}^{T+1}=\left\{\begin{array}{c}{Y}_{i,p}^{T} if within limits\\ {l}_{b} if {Y}_{i}^{T}<{l}_{b }\\ { u}_{b } if {Y}_{i}^{T}>{u}_{b}\end{array}\right.$$

The implementation of this mechanism is illustrated in the pseudo-code provided below.

**Figure:**
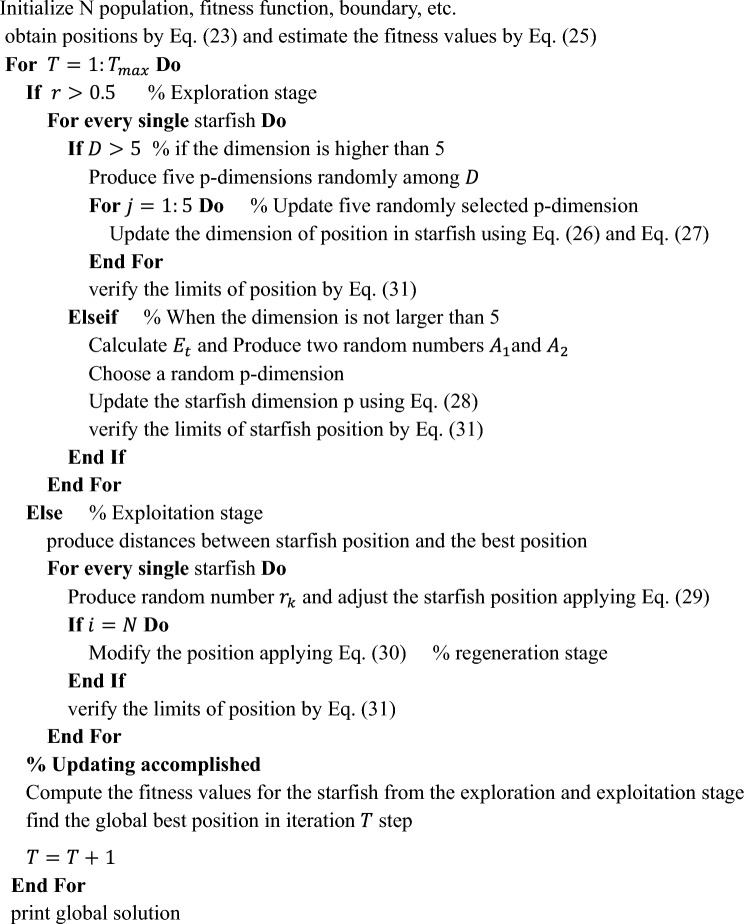
**Pseudo-code of the SFO approach**

#### Puma optimization approach

**Fig. 2:**
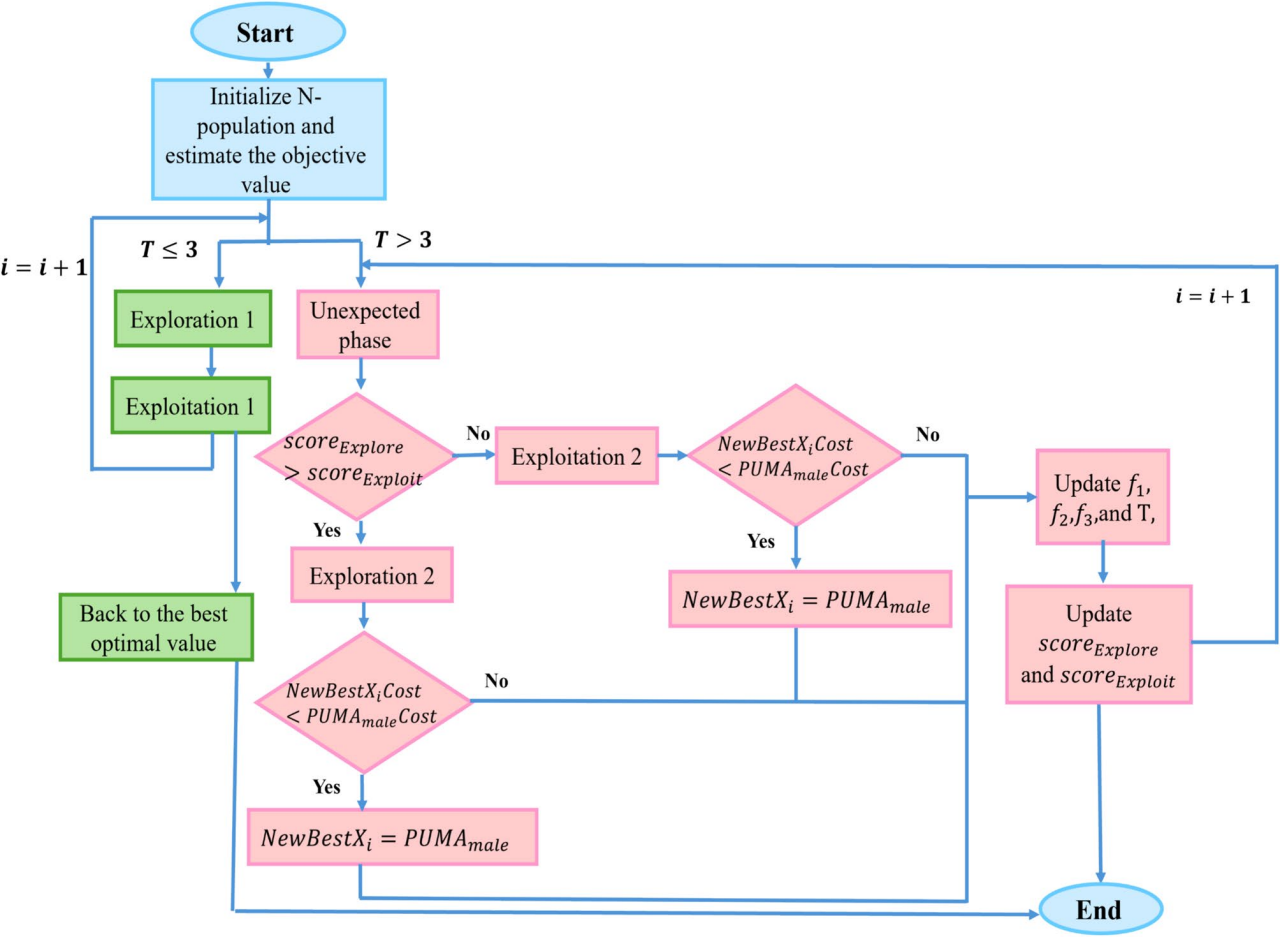
PO flowchart.

The PO is a nature-based metaheuristic that draws on the hunting strategies and adaptive behaviors of pumas, particularly their ambush tactics and territorial instincts. This algorithm models these traits through dynamic phase transitions and intelligent scoring mechanisms, enabling a balanced discovery and refinement of the space solution. By mimicking such adaptive behaviors, PO aims to efficiently locate global optima while minimizing the probability of untimely converging on non-global optima^48^. In this framework, the optimization landscape is conceptualized as the puma’s territory, where candidate solutions are represented as female pumas. The best-performing solution in the population is designated as the dominant male puma, exerting influence over the movement and decision-making of the others. This hierarchical structure and behavioral modeling are illustrated in Figure 2.

- Phases of Operation

i. Exploration Phase 1:
  Imitates the behavior of pumas foraging for sustenance in unexpected environments.
  Concentrates on producing a diversity of solutions to encompass a broad spectrum of space for solutions.

ii. Exploitation Phase 1
  Pumas return to known successful foraging sites.
  The finest solutions discovered thus far should be enhanced and refined.

- Phase Change process

i. Unexperienced Phase

- Exploration and exploitation are carried out concurrently during the first iterations to collect data on the solution domain.

Key Equations:

Exploration Score

32 $${S}_{Explore}=\left({PF}_{1}*{f1}_{Explore}\right)+\left({PF}_{2}*{f2}_{Explore}\right)$$

Exploitation Score

33 $${S}_{Exploit}=\left({PF}_{1}*{f1}_{Exploit}\right)+\left({PF}_{2}*{f2}_{Exploit}\right)$$

Depending on how well they perform, these scores dictate which step to proceed with.

ii. Experienced Phase
iii. The algorithm selects just one stage (exploration or exploitation) after several rounds, depending on which has done better.

Key Equations:

Cost enhancement for Exploitation

34 $${f1}_{exploit}={PF}_{1}*\left(\frac{{C}_{exploit\_old}-{C}_{exploit\_new}}{{T}_{exploit}}\right)$$

Diversity Component

35 $${f}_{3t}^{exploit}=\left\{\begin{array}{c}if opted, {f}_{3t}^{exploit}=0 \\ otherwise, {f}_{3t}^{exploit}+{PF}_{3}\end{array}\right.$$

This phase transition mechanism aims to avoid the optimization approach from getting stuck in local optima by allowing adaptive movement between exploration and exploitation phases.

The SFO and PO approaches are employed individually to address the introduced problem. The motivation behind using two separate algorithms is to perform a comparative evaluation of their effectiveness. SFO is chosen for its strong exploration ability, which helps to broadly investigate the solution space and avoid local minima, while PO is selected for its efficient exploitation capability, enabling rapid convergence to high-quality solutions. By applying both algorithms separately to the same optimization problem, we are able to highlight their respective strengths and limitations and thereby provide deeper insight into which algorithm is more suitable for addressing the nonlinear and multimodal nature of DG and EVCS allocation in distribution networks.

#### Particle swarm optimization

**Fig. 3:**
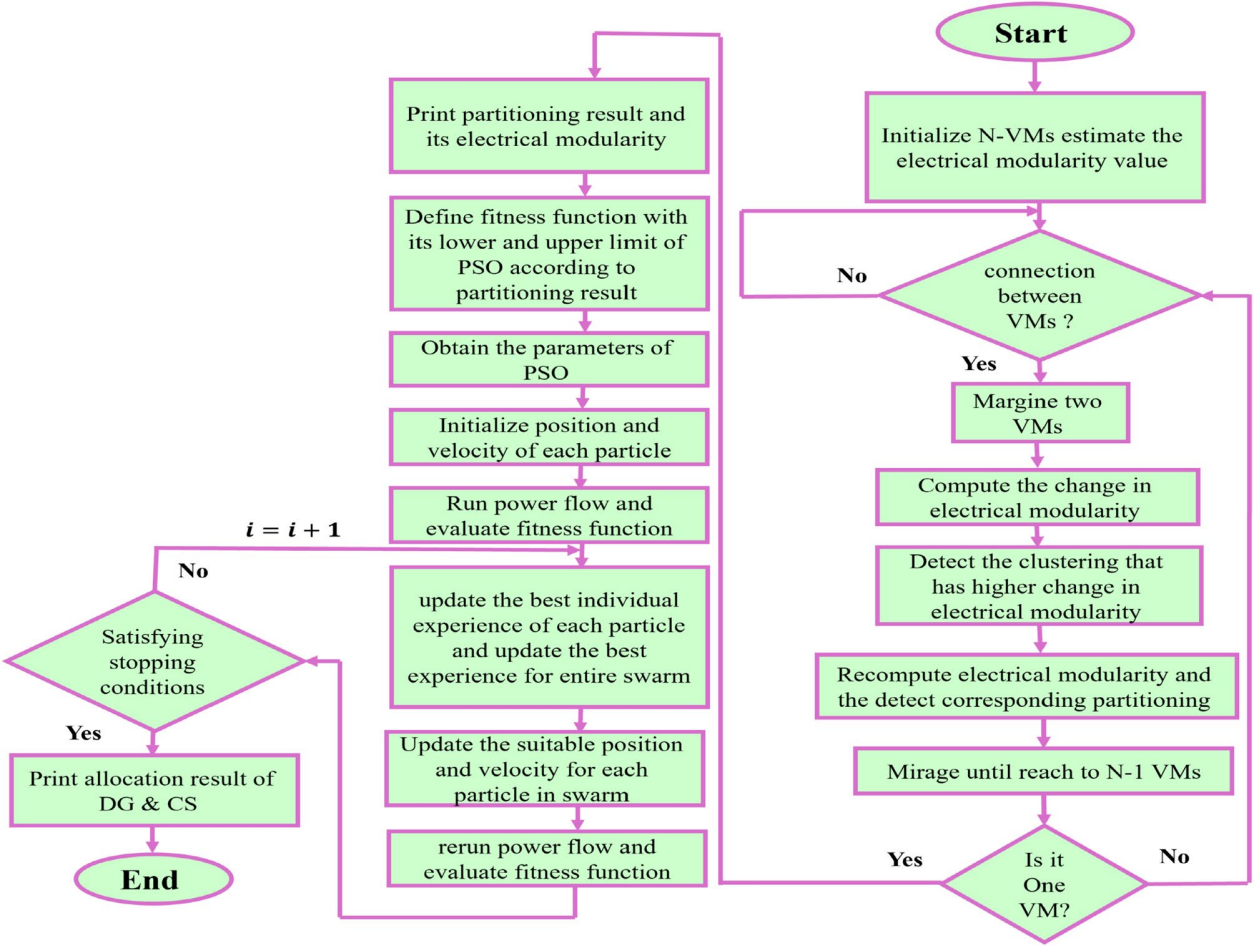
Modified NFA and PSO flowchart.

The PSO approach, introduced in the last century by Kennedy and Eberhart, is based on social behaviors observed in the swarming behavior of fish and birds. In this method, each solution, mentioned as a “particle” explores the space of search by adapting its location and speed based on both its individual solution of best-known (personal best) and the identified by the swarm are the best of global. This social cooperation enables the swarm to effectively converge toward optimal or near-optimal solutions through iterative learning and information sharing^49^. The net workflow of the PSO approach is depicted in Figure 3. In this research, PSO is implemented to detect the best allocation of EV-CSs and resources within clustered PDNs^1,55^. The algorithm is configured to solve a constrained multiple-objective function, aiming to lessen energy losses and voltage instability. The search space dimensionality is defined by four decision variables per virtual microgrid, corresponding to the location and size of each EV-CS. In addition, the capacity and power factor of the resource, result in a search space of 4×N dimensions, where N is the identified communities number.

- i. Benchmark system

#### IEEE 33-bus PDN

**Fig. 4:**
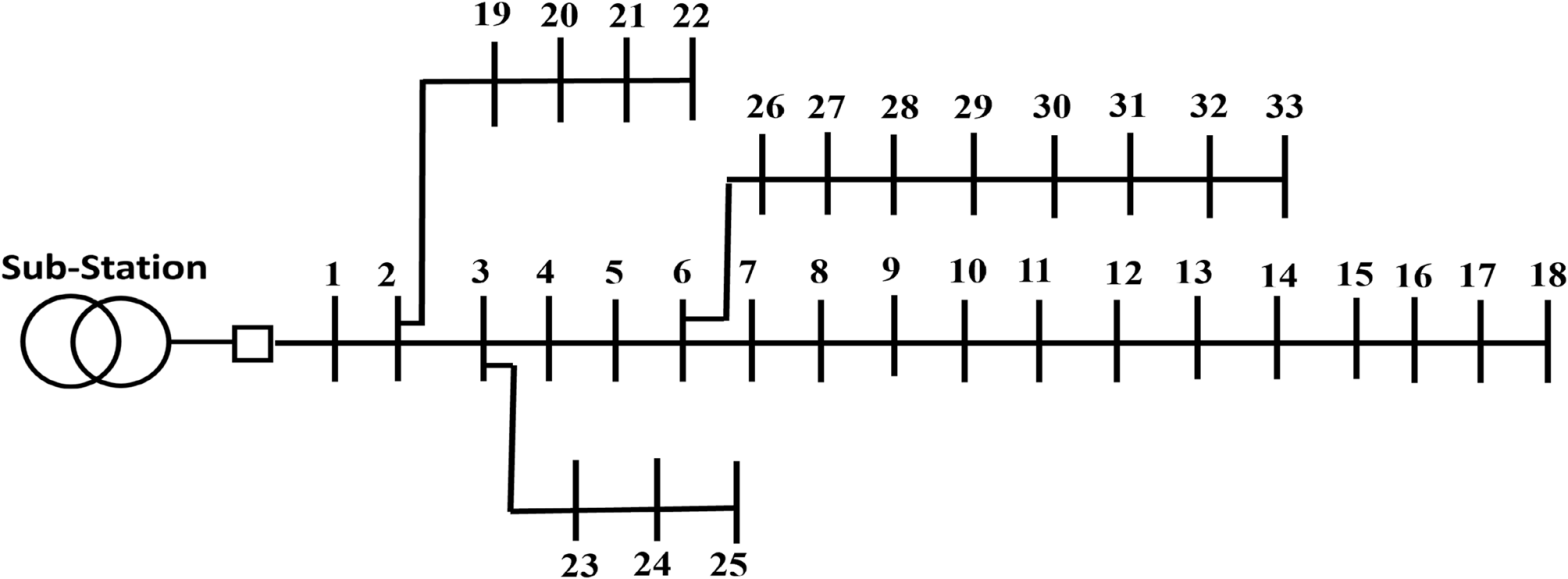
One-line schematic of 33-bus PDN.

The benchmark radial PDN used in this study involves 33 buses and 32 distribution lines, as illustrated in Figure 4. The network operates at 12.66 kV of rated voltage, with a MVA base of 100^56^. The overall real and imaginary demands of PDN are 3.72 MW and 2.30 MVAR, respectively. Under original case conditions, the power loss is 0.211 MW, while the lowest absolute voltage and the SI are 0.9038 and 0.6681 p.u., respectively.

#### IEEE 118-bus system

**Fig. 5:**
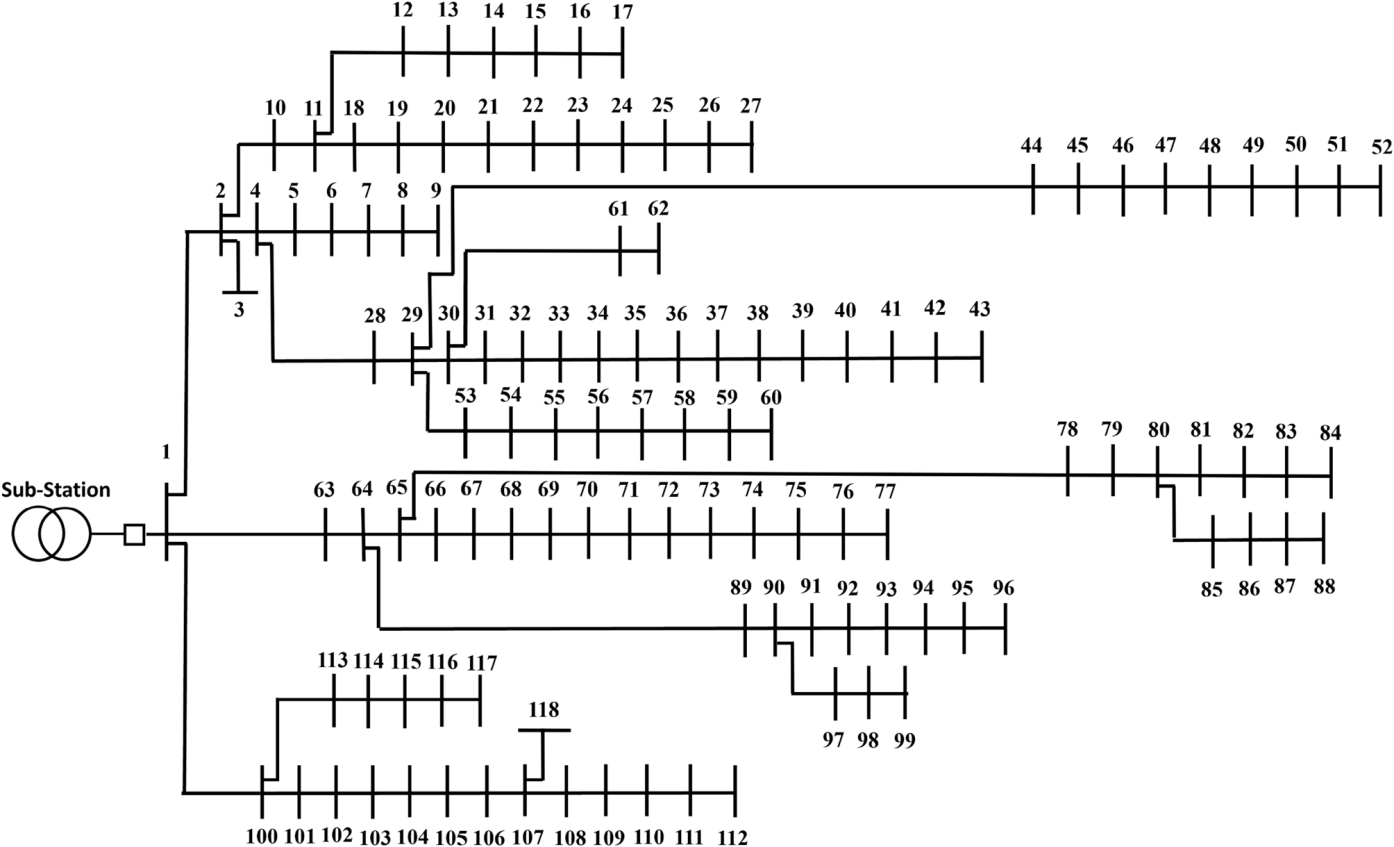
One-line schematic of 118-bus PDN.

The second radial PDN analyzed in this study includes 118 buses and 117 distribution lines, as illustrated in Figure 5. The base voltage level is 11 kV with a MVA base of 100. The summation of loads in the PDN amounts to 22.71 MW of real power and 17.04 MVAR of imaginary power. Under the base case scenario, the system exhibits active power losses of 1.638 MW. Additionally, the minimum bus voltage absolute and the SI are recorded as 0.8361 and 0.6256 p.u., respectively^57,58^.

## Converting benchmark system into real-time system

### Demand-side modeling and daily load profiles

The load demand in a distribution system exhibits different responses to modifications in system voltage. Therefore, the real performance of the load is not exhibited by a fixed value of current, impedance, or power, but instead indicates a compound combination. A variety of load types exist within the delivery network, including residential (Home), industrial (Factory), and commercial (retail) categories. In this paper, a static load profile is used to depict the real and imaginary power demand. Additionally, in each VM in the tested system will be assumed as a zone of this type of load. This research introduces a modeling approach of static load, where the active and reactive power consumption are formulated via a polynomial-based framework. The Model of Polynomial Load^59^ is represented as follows

36 $${p}_{d}={p}_{d0}*\left[{a}_{p}+{a}_{i}\left(\frac{v}{{v}_{o}}\right)+{a}_{z}{\left(\frac{v}{{v}_{o}}\right)}^{2}\right]$$

37 $${q}_{d}={q}_{d0}* \left[{aa}_{p}+{aa}_{i}\left(\frac{v}{{v}_{o}}\right)+{aa}_{z}{\left(\frac{v}{{v}_{o}}\right)}^{2}\right]$$

where the coefficients $a$ and $aa$ present the load component proportions which they are fixed power (p), current (i), and impedance (z) with the constraints

38 $${a}_{p}+{a}_{i}+{a}_{z}=1$$

39 $${aa}_{p}+{aa}_{i}+{aa}_{z}=1$$

In this study, there are specific coefficients obtained for residential, commercial, and industrial loads as revealed in Table 1^60^.

**Table 1:** Proportional composition of various types of loads.

Composition			
Active power			
	Residential	Commercial	Industrial
${a}_{p}$	0.13	0.04	0.83
${a}_{z}$	0.24	0.16	−0.07
${a}_{i}$	0.62	0.80	0.24
Reactive power			
	Residential	Commercial	Industrial
${a}_{p}$	0.50	0.84	0
${a}_{z}$	2.44	3.26	1
${a}_{i}$	−1.94	−3.10	0

## Results and discussion

### Applying the modified newnan fast algorithm

**Fig. 6:**
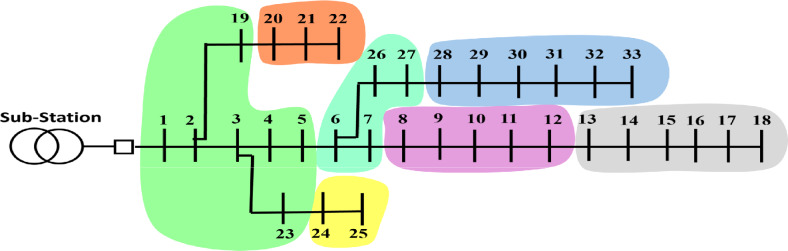
Results of partitioning for 33-bus PDN.

After applying the Modified NFA using both the topological adjacency matrix and the ECS matrix, successful network partitioning was achieved for all benchmark PDNs, including the 33-bus and 118-bus. This partitioning process facilitated the decomposition of each test system into distinct and electrically cohesive VMs, enabling more effective localized planning and control. The resulting VM structures for the PDN are shown in Figure 6.

**Fig. 7:**
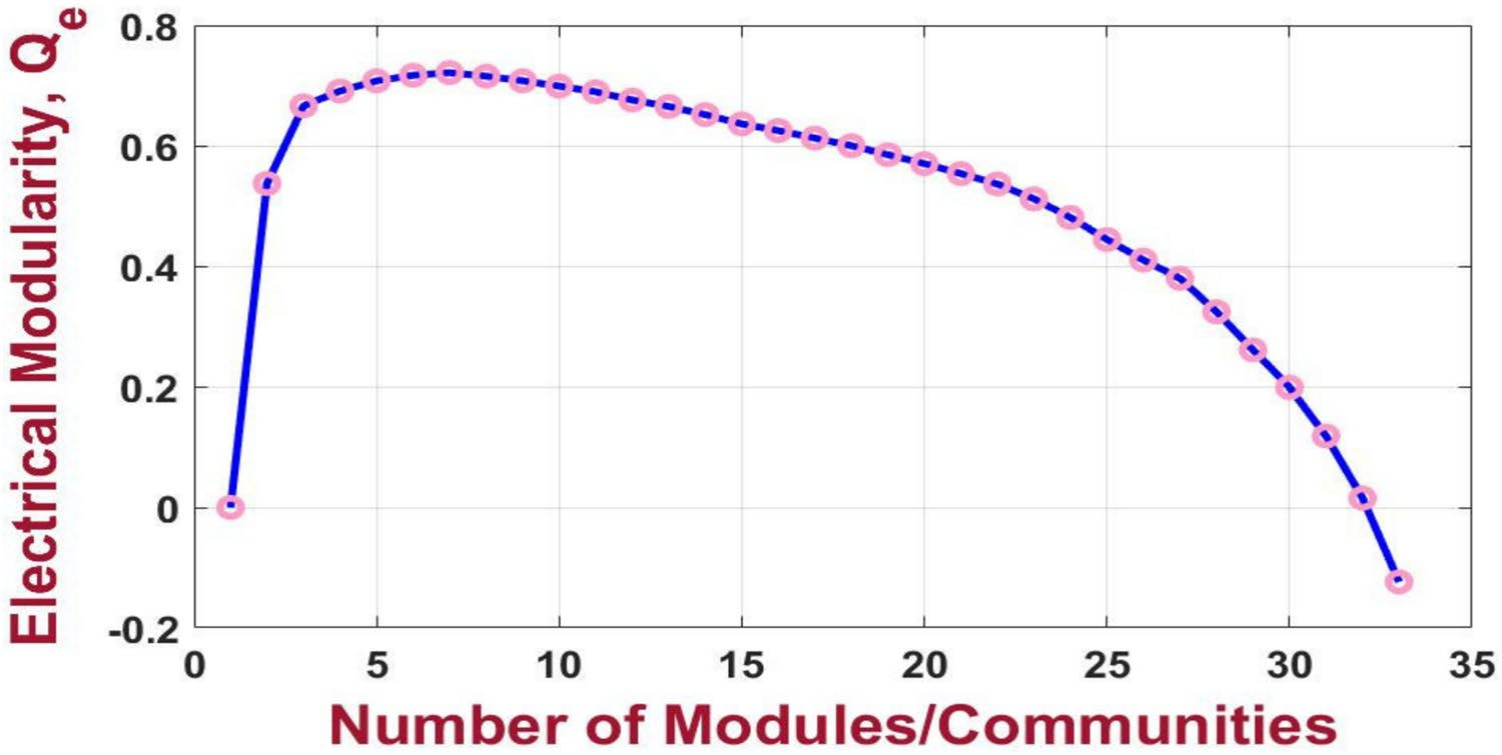
Electrical modularity vs the number of modules resulting from the modified NFA for 33-bus PDN.

For the 33-bus PDN, the highest electrical modularity is achieved when the network is partitioned into seven communities, as detailed in Table 2. The resulting electrical modularity value is 0.724, indicating a well-defined and electrically cohesive community structure. This demonstrates an improvement over the approach proposed in^38^, where the electrical modularity for the same test system is reported as 0.71 as depict in Figure 7. The enhanced modularity highlights the significance of the presented partitioning strategy in defining the underlying electrical interdependencies within the network.

**Table 2:** Clustering results of 33-bus PDN.

VM number	Bus number
1	1–5,19,23
2	28–33
3	20–22
4	24–25
5	8–12
6	13–18
7	6–7,26–27

**Fig. 8:**
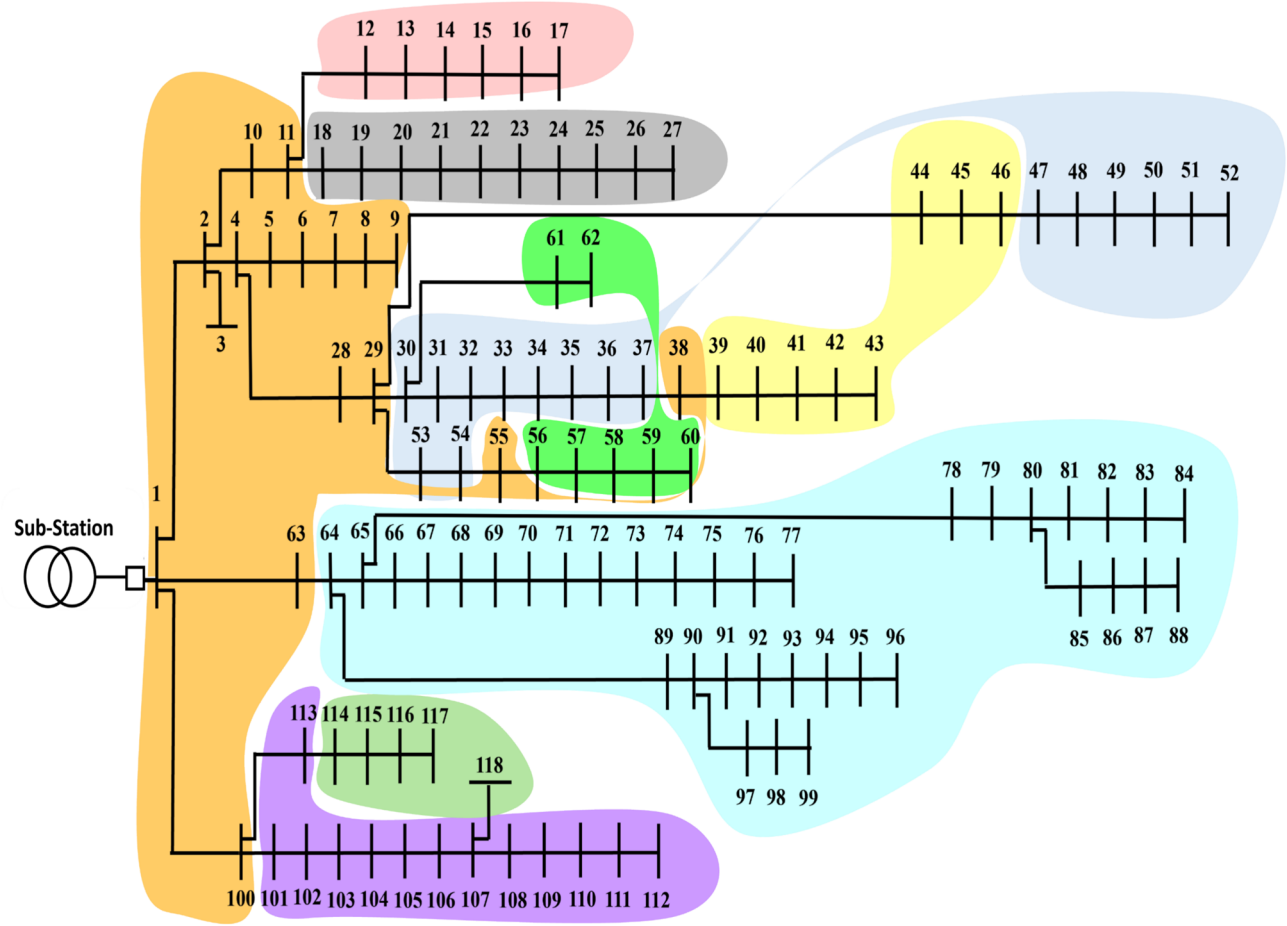
Result of partitioning for 118-bus PDN.

**Fig. 9:**
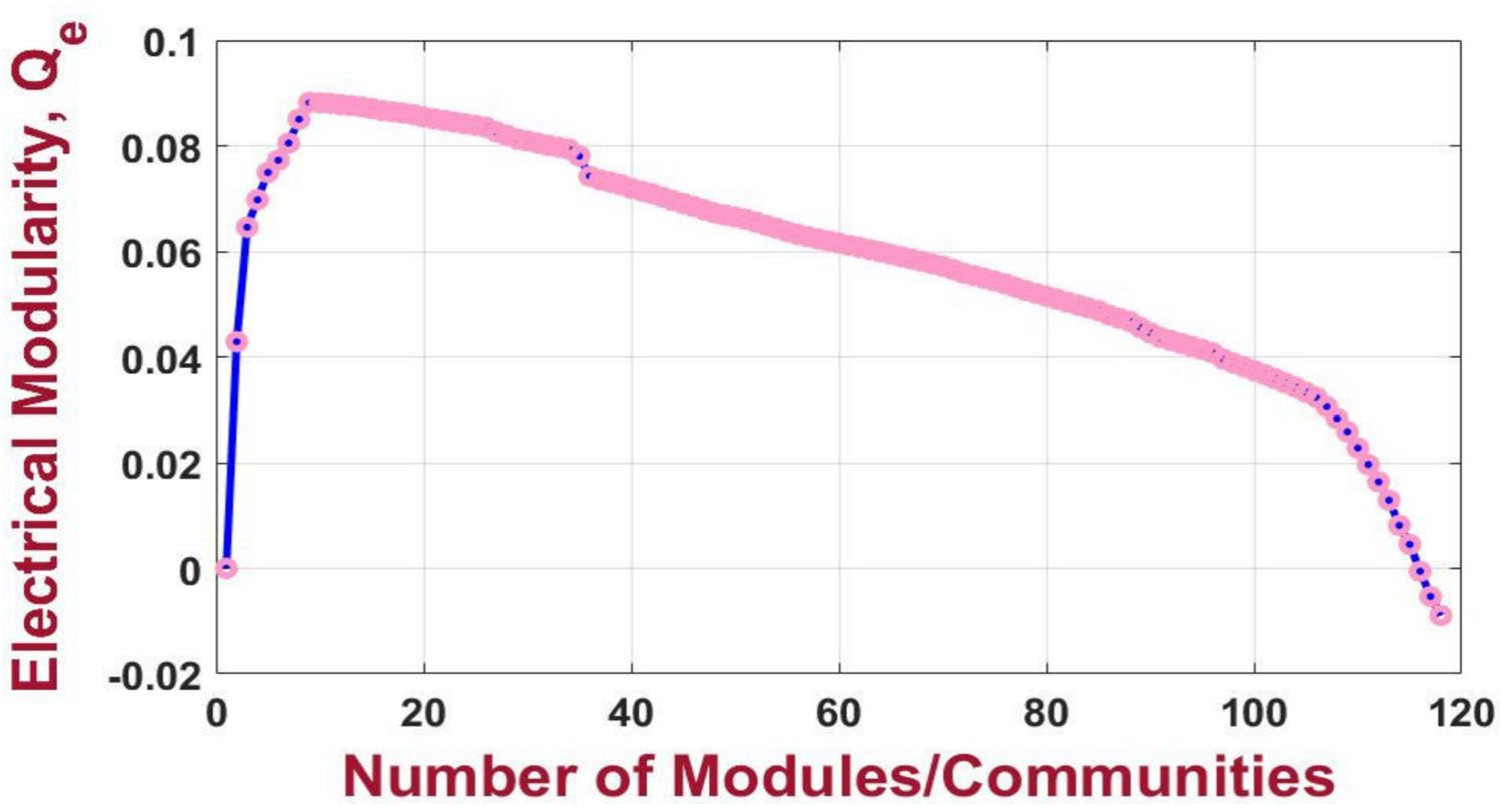
Electrical modularity vs the number of modules resulting from the modified NFA for 118-bus PDN.

The partitioning of the 118-bus PDN applying the proposed method, achieves its maximum electrical modularity when the PDN is clustered into nine VMs as in Figure 8. For the 118-bus PDN, the highest electrical modularity is achieved when the network is partitioned into nine communities, as detailed in Table 3. The corresponding modularity value is 0.089, as shown in Figure 9, indicating a relatively lower degree of internal electrical cohesion compared to the smaller test systems. This lower modularity can be attributed to the increased size and complexity of the network, which may reduce the distinctiveness of community boundaries in terms of electrical interdependence.

**Table 3:** Partitioning results of 118-bus PDN.

VM number	Bus number
1	1–11, 28–29, 38, 55, 63,100
2	114–118
3	12–17
4	56–62
5	101–113
6	64–99
7	18–27
8	30–37,47–54
9	39–46

### Allocation of CSs and DGs results

**Fig. 10:**
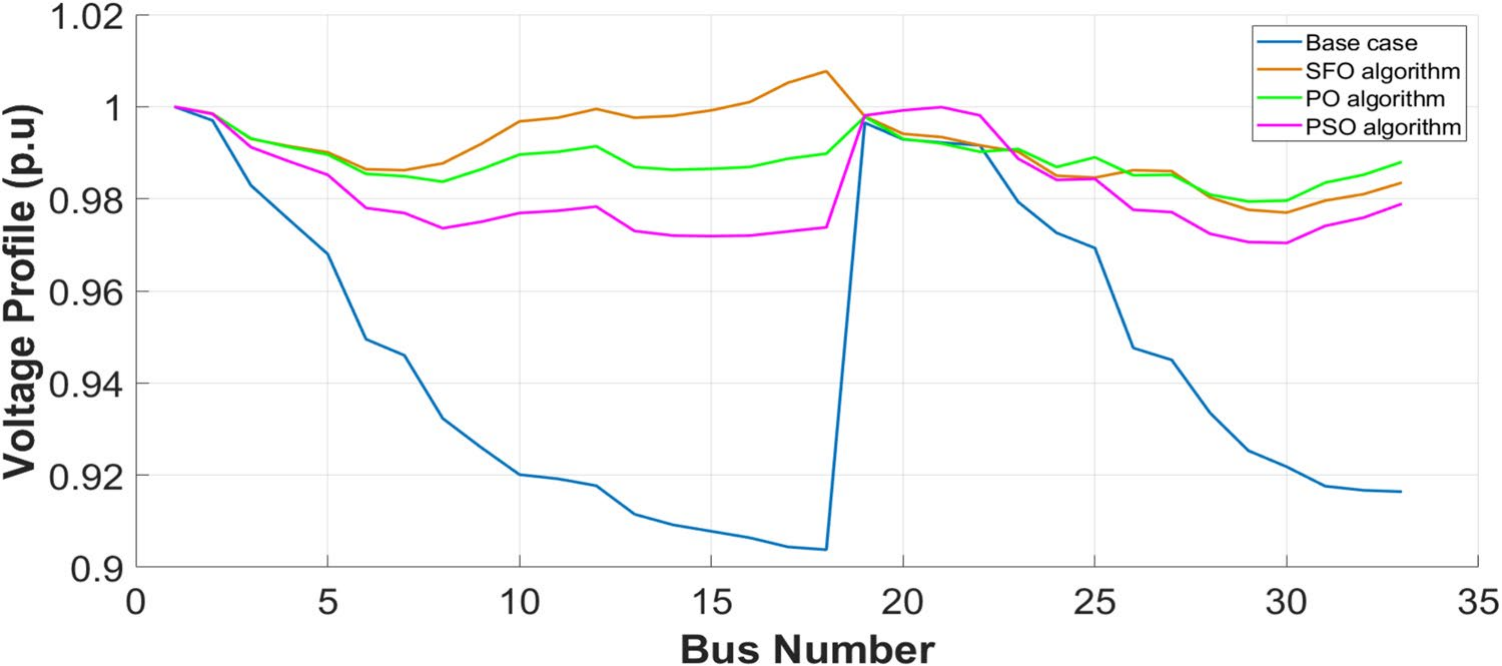
Voltage profile for 33-bus PDN.

The optimum location and capacity of EV-CSs and resources are determined depending on the VM partitioning results, with each VM assigned one DG and one EV-CS. These resources are strategically allocated at the bus exhibiting the lowest SI within each VM to enhance local voltage support. The optimization is performed using the SFO and PO, and their performance is benchmarked against the conventional PSO algorithm for the 33-bus PDN, as illustrated in Table 4. Thus, the results demonstrate that the presented algorithms (SFO and PO) outperform PSO in terms of both SI enhancement and decline in power loss, thereby validating the importance of the recommended strategy. The results presented in Table 4. demonstrate a notable reduction in loss, approximately 82% relative to the original case scenario. Furthermore, the bottom bus voltage in magnitude increases to 0.979 p.u as illustrated in Figure 10, which in turn enhances the SI to 0.927 p.u. Among the tested optimization algorithms, the PO exhibits the fastest convergence rate, outperforming both the SFOA and PSO in terms of execution time, thereby confirming its computational efficiency and effectiveness in solving the joint allocation problem. Additionally, the proposed techniques were compared with other algorithms such as comprehensive learning PSO (CLPSO), Differential evolution (DE), and shuffled frog leaping (SFL) algorithms.

**Table 4:** Allocation results of DGs & CSs for IEEE 33-bus PDN.

**IEEE 33-bus System**										
**Optimization Algorithm**	EVCS Location	EVCS size (MW)	DG Location	DG size (MVA)	Power Factor		${TP}_{loss}$ (kW)	${\mathrm{SI}}_{\mathrm{min}}$ $(p.u)$	${V}_{min} (p.u)$- Bus No.	Time (sec)
**Base case**							211	0.668	0.9038–18.9038	
**SFO**	2,8,13,22,24,6,28	0.2500 0.2500 0.2500 0.2500 0.2500 0.2500 0.2500	5,12,18,21,25,27,33	0.1560 0.9907 0.5499 0.1829 0.4111 0.5829 0.7550	0.9115 0.9439 0.9447 0.9500 0.9288 0.8804 0.8000		37.791	0.9098	0.9770–30.9770	436.44
**PO**	2,8,13, 22,24,26,28	0.2500 0.2500 0.2500 0.2500 0.5000 0.2500 0.2500	5,12,18,21,25, 27,33	0.1001 0.9890 0.3100 0.1002 0.8000 0.5152 1.0000	0.9500 0.9094 0.9290 0.9500 0.9500 0.8006 0.9500		38.290	0.9272	0.9794–29.9794	419.06
**PSO**	19,8,13, 22,24,6, 28	0.2500 0.2500 0.2500 0.2500 0.2500 0.2500 0.2500	5,12,18,21,25, 27,33	0.1124 0.9100 0.2497 0.6009 0.5000 0.3009 0.8800	0.9114 0.9439 0.9447 0.9500 0.9288 0.8804 0.8000		45.681	0.886	0.9704–30.9704	507.77
**DE**^14^	2,8,13, 22,24,6,28	0.2500 0.2500 0.2500 0.2500 0.2500 0.2500 0.2500	5,12,18,21,25, 27,33	0.1000 0.9040 0.5499 0.3828 0.4060 0.5828 0.7000	0.9111 0.9404 0.9500 0.9500 0.9287 0.8800 0.8000		39.7667	0.8948	0.9730–31.9730	469.53
**CLPSO**^14^	19,12,13, 22,24,6, 33	0.2500 0.2500 0.2500 0.2500 0.2500 0.2500 0.2500	5,11,8,21,25,27,32	0.1102 0.8998 0.3340 0.5492 0.4102 0.974 0.5236	0.9500 0.8000 0.9500 0.8000 0.8000 0.9500 0.9500		46.569	0.868	0.9650–33.9650	454.51
**FLS**^15^	19,8,13, 21,24,6, 32	0.2952 0.2518 0.2519 0.2628 0.2521 0.2524 0.2539	5,12,18,22,25,27,33	0.2919 0.6948 0.5415 0.2755 0.1915 0.8975 0.6945	0.9240 0.9242 0.9386 0.9307 0.9278 0.8640 0.8094		40.2858	0.8940	0.9743–30.9743	1672.8

**Fig. 11:**
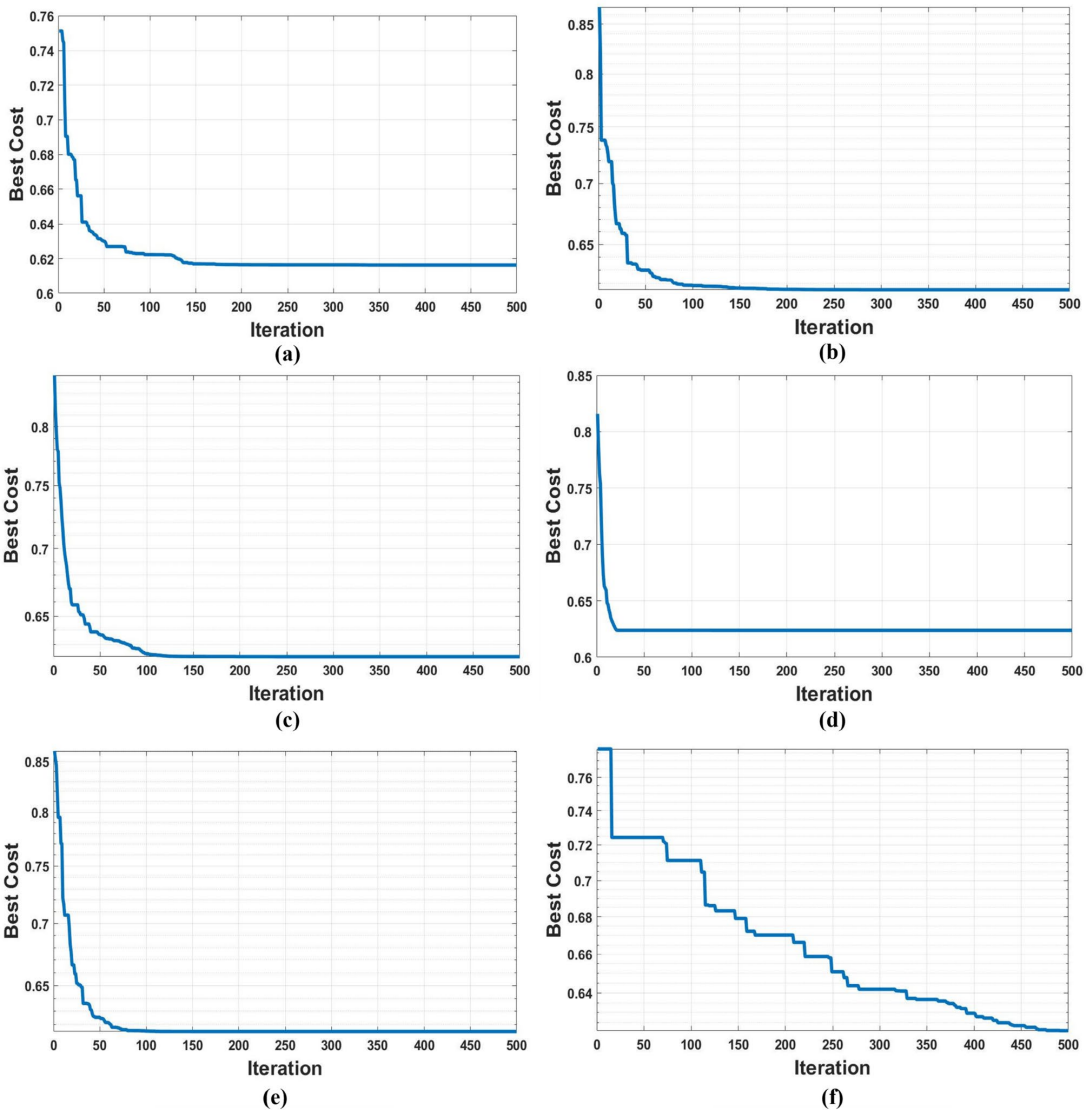
Convergence curve of algorithms for IEEE 33-bus PDN: (**a**) SFO, (**b**) PO, (**c**) PSO, (**d**) CLPSO, (**e**) DE, (**f**) SFL.

The convergence analysis, as shown in Figure 11, revealed smooth profiles with low variance across several independent runs, indicating stable behavior of all algorithms. Furthermore, the feasibility rate consistently reached 100%, confirming the robustness of SFO and PO when applied to the high-dimensional, non-convex DG and EV-CS co-placement problem. Compared with the algorithms in^14,15^, the proposed SFO and PO exhibit superior convergence speed and solution stability, which is crucial for real-time planning. While all algorithms achieve acceptable loss minimization, the reduced variance and faster execution of SFO/PO provide an advantage in large-scale systems. Figure 11 presents convergence curves, indicating smooth and consistent convergence without premature stagnation in which the Figure 11 (a) shows the convergence curve of SFO which reaches to optimum value after 170 iterations also Figure 11 (b) illustrates the PO convergence curve that achieving its optimal value after approximately 180 iterations with its lowest score value, Figure 11 (c) present PSO approach which spend 130 iterations to reach the score 0.622. Figure 11 (d) shows the CLPSO algorithm’s fastest convergence for approximately 20 iterations to reach the objective value of 0.623. Finally, Figures 11 (e) and (f) present the techniques DE and SFL, which both reached 0.62, but the SFL had the lowest speed of convergence despite the best optimal values.

**Fig. 12:**
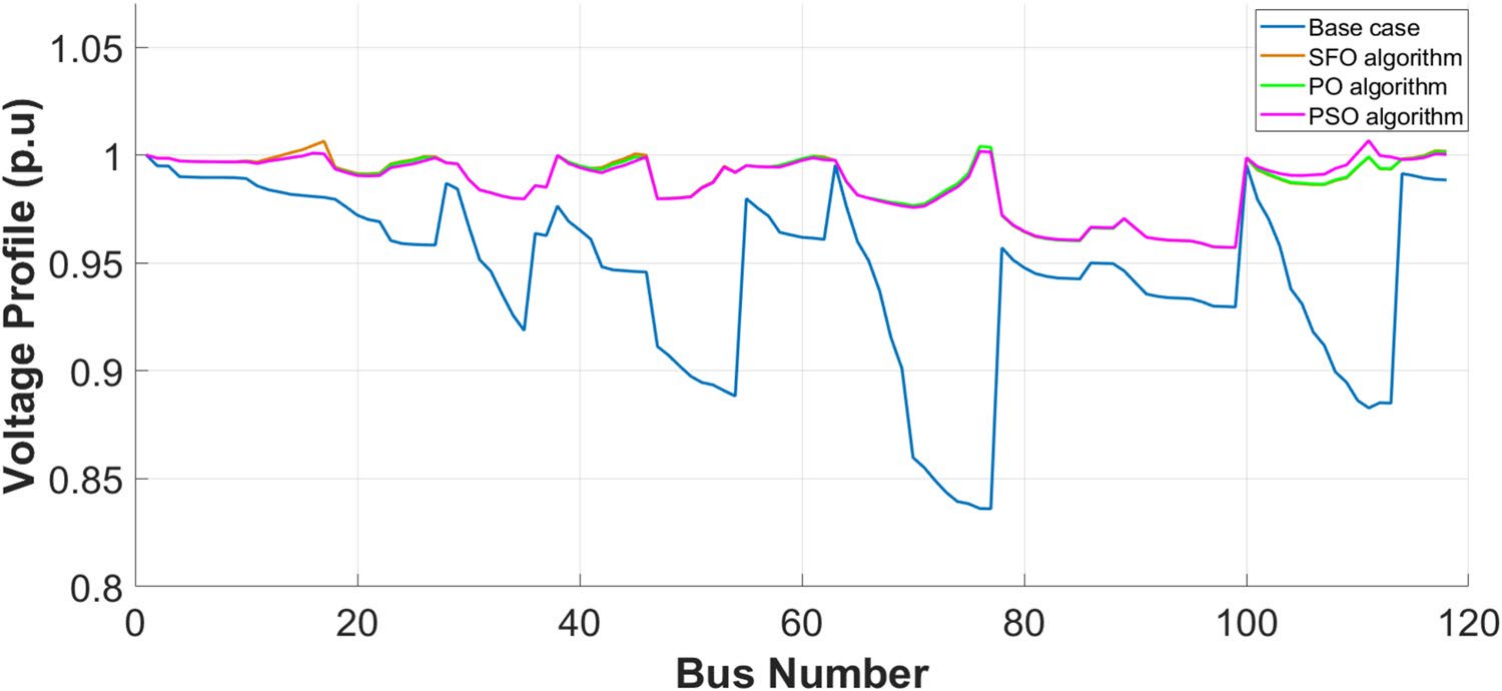
Voltage profile for IEEE 118-bus PDN.

As illustrated in Table 5., the IEEE 118-bus PDN also demonstrates a substantial reduction in active power losses, ranging from 68% to 69%, depending on the optimization algorithm used. In addition, significant improvements are observed in both the minimum bus voltage magnitude, as shown in Figure 12, and the SI. These output data verify the robustness and scalability of the suggested allocation strategy, validating its effectiveness across different network sizes. Moreover, the performance achieved surpasses that reported in several related studies^55,61,62^, highlighting the advantages of the presented approach in enhancing PDN efficiency and stability.

**Table 5:** Allocation results of DGs & CSs for IEEE 118-bus PDN.

**IEEE 118-bus PDN**									
**Optimization Algorithm**	EVCS Location	EVCS size (MW)	DG Location	DG size (MVA)	Power Factor	${\mathrm{TP}}_{\mathrm{loss}}$ (kW)	${\mathrm{SI}}_{\mathrm{min}}$ $(p.u)$	${V}_{min} (p.u)$- Bus No.	Time (sec)
**Base case**	-	-	-	-	-	1298.1	0.569	0.8688 −77	
**SFO**	100, 12, 27, 54, 46, 60, 77, 101, 118	0.4158 0.2667 1.1659 0.2641 0.2520 0.2221 0.2502 0.2260 0.2000	38, 17, 26, 53, 45, 61, 76,111,117	2.2535 1.1488 0.8623 2.9407 1.1074 1.0685 3.0445 3.4359 0.6050	0.8092 0.8558 0.8281 0.8212 0.9248 0.8000 0.8668 0.8092 0.9351	407.3758	0.8398	0.9571–99.9571	3611.5
**PO**	2, 17, 27, 54, 46, 62, 77, 101, 118	0.2001 0.2049 0.2935 0.2000 0.2000 0.2000 0.2229 0.2002 1.7449	38, 16, 26, 53, 45, 61, 76,111,117	2.4477 0.9380 0.8658 2.8659 0.9864 1.0567 3.0224 3.4485 0.5542	0.8000 0.8814 0.8014 0.8000 0.9009 0.8000 0.8588 0.8000 0.8001	399.3547	0.8399	0.9571–99.9571	4090.2
**PSO**	2,17,18,54,39,62,77,113,118	0.2000 0.2000 1.0350 0.2000 0.2000 0.2000 0.2000 0.2968 0.2000	38,16, 27,53, 46,61, 76,111, 117	2.6960 0.9321 0.8146 2.86800.7616 1.01602.9840 4.0000 0.4762	0.8079 0.8000 0.8062 0.8000 0.8585 0.9500 0.8000 0.8000 0.8237	414.5212	0.840	0.9572–99.9572	3734.3
**DE**^14^	2, 17, 27, 54, 46, 62, 76, 101, 118	0.2000 0.2000 1.0543 0.2000 0.2000 0.2000 0.2000 0.2000 0.8611	38,16, 26,53 45,61 77,111, 117	2.4527 0.9246 0.8595 2.8636 0.9831 1.0505 2.9937 3.4405 0.4948	0.8000 0.8797 0.8000 0.8000 0.9006 0.8371 0.8560 0.8000 0.8201	409.3444	0.8396	0.9571–99.9571	4661.1
**CLPSO**^14^	100,17,27,54,39,62, 64,110, 118	0.2000 0.3187 1.6951 0.2000 0.2002 0.2276 0.2001 0.2001 1.5026	38,16, 26,53, 46,61, 77,111, 117	2.6280 1.0237 0.8751 2.8708 0.7456 1.0903 2.7623 3.5745 0.4991	0.8318 0.9457 0.8049 0.8000 0.8471 0.8381 0.8403 0.8000 0.8135	412.3395	0.8377	0.9565–99.9565	2799.9
**SLF**^15^	9,17,27,54,46,56,77,110,118	0.2265 0.2328 0.9972 0.2078 0.4110 0.6293 0.2798 0.2169 0.9703	55,16, 26,53, 45,61, 76,111, 117	2.4882 1.0517 0.8822 2.8642 1.1684 1.4157 3.1784 3.5616 0.4172	0.8126 0.8536 0.8191 0.8058 0.9176 0.9164 0.8610 0.8013 0.8145	433.9962	0.8415	0.9576–99.9576	14529.9

In this study, the main contribution is the deployment of two recently developed metaheuristics SFO and PO—for optimal allocation of DGs and EV-CSs. Unlike conventional PSO, which was included as a benchmark, SFO leverages regeneration and preying behaviors to enhance the balance between exploration and exploitation, while PO adapts puma hunting strategies to dynamically shift between global and local search. These features help overcome the premature convergence and limited adaptability typically observed in PSO. the results highlight that SFO and PO not only provide superior solution quality in terms of minimizing total losses and enhance voltage profiles, but also demonstrate higher robustness across different trials and faster convergence toward near-optimal solutions.

**Fig. 13:**
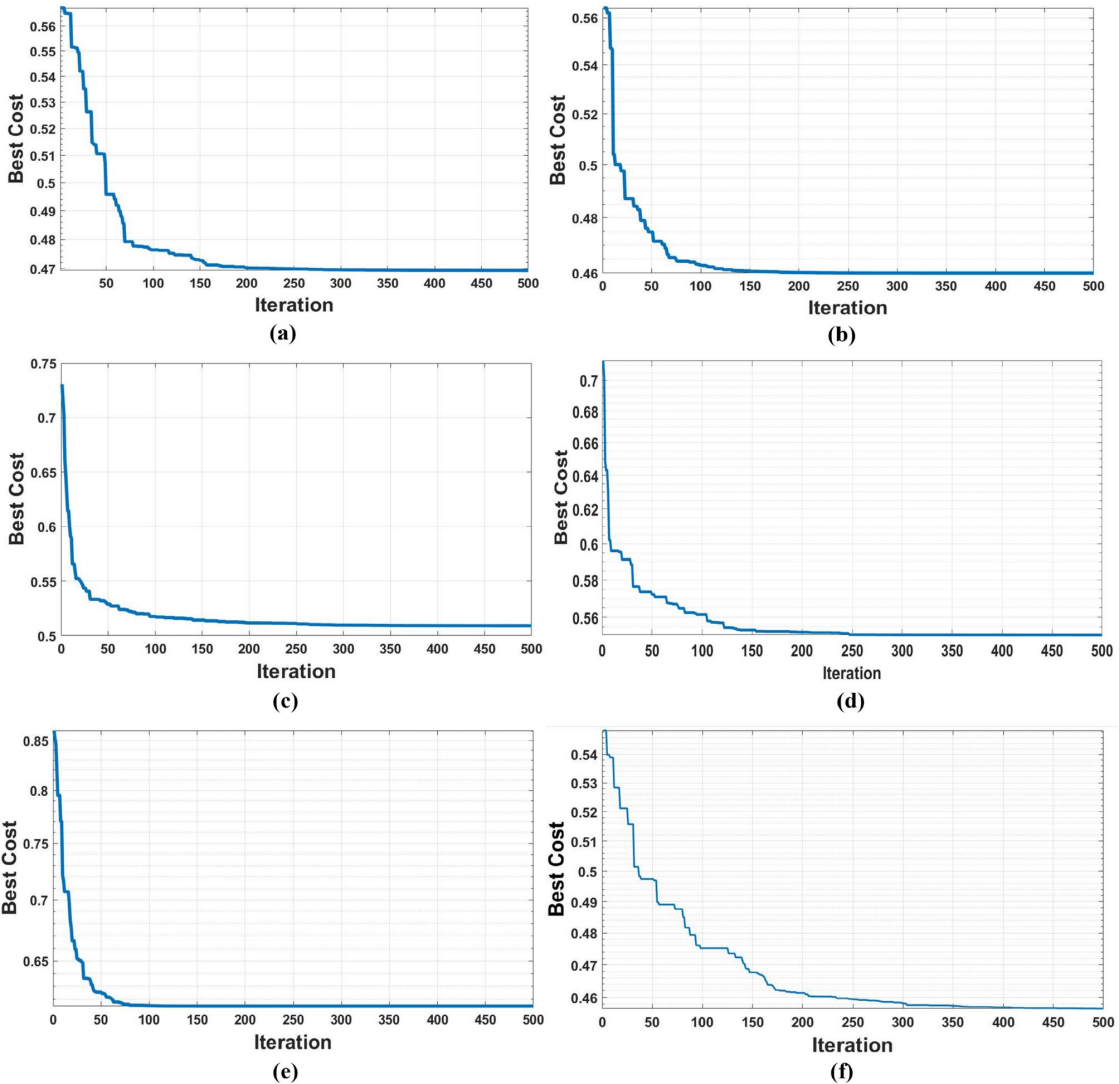
Convergence curve of algorithms for IEEE 118-bus PDN: (**a**) SFO, (**b**) PO, (**c**) PSO, (**d**) CLPSO, (**e**) DE, (**f**) SFL.

Although the proposed SFO and PO algorithms provide robust solutions for DG and EV-CS co-placement, their computational time increases with network size as shown Table 4 and Table 5.. The study was implemented in MATLAB R2018a using an M-file script on a personal computer (Acer Aspire A515-54G laptop, Intel Core i5 processor, 8 GB RAM, Windows 10). Therefore, for the same computer with its same specifications used for large systems such as the IEEE 118-bus network, a larger population and more iterations are needed to explore the expanded search space effectively. Consequently, direct real-time application is limited. However, the integration of network partitioning (modified Newman Fast Algorithm) and local optimization within clusters, along with reduced population sizes or surrogate models, can enhance computational efficiency and enable near real-time applicability. The convergence curves are shown in Figure 13. In Figure 13 (a), the convergence curve of SFO indicates that time increases due to the increase in system scale. Hence, the best score is achieved at iteration 290. However, the fastest algorithm to reach the best score is PO, as demonstrated in Figure 13 (b). The PSO curve shown in Figure 13 (c) illustrates that the best cost is 0.51, achieved after 350 iterations. Furthermore, for CLPSO and DE, they attain their best cost at around 300 iterations, as depicted in Figure 13 (d) and Figure 13 (e). respectively. However, in Figure 13 (f). provides its curve that achieves the best fitness value after approximately 415 iterations by SFL.

Beyond active power loss and voltage profile improvement, the proposed methodology was evaluated using additional metrics to quantify system robustness and operational resilience. Table 6. summarizes SI, LLI%, VD, and maximum EV hosting capacity for both IEEE 33- and 118-bus systems when the EV-CSs are loaded by 20% of their capacity. For both PDNs, the allocation results show that the increase in loading increased the LLI% and VD, so the SI decreased. For the 33-bus PDN, the maximum of LLI% is for the proposed SFO approach when the loading increases, which leads to a higher percentage of maximum EV hosting capacity. While the lowest VD is introduced by the PO technique, which leads to the highest SI. However, the effect of a 20% raising in CS for the 118-bus PDN has an insignificant impact on the metrics values due to the low capacity of EV-CS that depends on the total number of EVs^54^*.*

**Table 6:** Performance metric under high EV penetration of both PDNs.

**System**	**Metric**	**Base Case**	**(SFO)**	**(SFO)** **(EVCS+20%)**	**(PO)**	**(PO)** **(EVCS+20%)**	**(PSO)**	**(PSO)** **(EVCS+20%)**
**IEEE-33**	${SI}_{min}$ (p.u)	0.668	0.9098	0.8967	0.9272	0.9129	0.886	0.8728
	${LLI\%}_{max}$	42.19%	21.69%	24.78%	22.03%	25.57%	22.48%	25.61%
	${VD}_{min}$ (p.u)	0.096	0.023	0.0265	0.0206	0.0244	0.0296	0.0338
	Maximum EV Hosting Capacity (kW)	-	-	140	-	116.5	**-**	74.28%
**IEEE-118**	${SI}_{min}$ (p.u)	0.569	0.844	0.8392	0.844	0.8393	0.841	0.8395
	${LLI\%}_{max}$	52.15%	24.63%	24.78%	24.61%	24.75%	24.7%	24.83%
	${VD}_{min}$ (p.u)	0.131	0.041	0.0431	0.041	0.043	0.042	0.043
	Maximum EV Hosting Capacity (kW)	-	-	520%	-	575%	-	600%

The three algorithms were executed several times. Table 7. presents statistical results obtained using the Wilcoxon test, highlighting high variance between PO and PSO, as well as between SFO and PSO. However, there is a small variance between PO and SFO, which confirms the stability of both PO and SFO for the PDNs.

**Table 7:** Wilcoxon test for both systems results.

**System**	**Algorithms Comparison**	**Wilcoxon Criteria**		
		${R}^{+}$	${R}^{-}$	$p$-value
**IEEE-33**	PO vs PSO	2	64	0.0029
	SFOA vs PSO	4	62	0.0068
	PO vs SFO	18	10	0.5781
**IEEE-118**	PO vs PSO	3	63	0.004882
	SFOA vs PSO	4	62	0.00585
	PO vs SFO	21	45	0.30957

Table 8. introduces the results for the proposed work comparing with other algorithms from several research papers. the best results achieved were from HHO and TLBO algorithms, which the losses reduction parentages are 89.05% and 89.02% respectively. However, the concept of partitioning the network does not achieve by these approaches. In addition, the other studies have not discussed the clustering aspect also they have a lower power losses reduction than the proposed techniques.

**Table 8:** Comparison between the paper results and the other results for IEEE 33-bus PDN.

**Algorithm**	${{\boldsymbol{T}}{\boldsymbol{P}}}_{{\boldsymbol{l}}{\boldsymbol{o}}{\boldsymbol{s}}{\boldsymbol{s}}}$**(kW)**	${{\boldsymbol{T}}{\boldsymbol{P}}}_{{\boldsymbol{l}}{\boldsymbol{o}}{\boldsymbol{s}}{\boldsymbol{s}}}$ **reduction %**	${{\boldsymbol{S}}{\boldsymbol{I}}}_{{\boldsymbol{m}}{\boldsymbol{i}}{\boldsymbol{n}}}\boldsymbol{ }({\boldsymbol{p}}.{\boldsymbol{u}})$	${{\boldsymbol{V}}}_{{\boldsymbol{m}}{\boldsymbol{i}}{\boldsymbol{n}}}\boldsymbol{ }({\boldsymbol{p}}.{\boldsymbol{u}})$	**Partitioning**
Base case	211	-	0.668	0.9038	-
SFO	37.791	82.08%	0.9098	0.9770	
PO	38.290	81.85%	0.9272	0.9794	
HHO^63^	32.3824	89.05%	0.9392	0.9845	
TLBO^63^	32.4586	89.02%	0.9391	0.9845	
BESA^64^	80.49	64.099%	0.777	0.9409	
CSA^64^	86.13	61.58%	0.7652	0.9382	
PO^65^	67.191	68.15%	0.8975	0.9785	
HGAPSO^61^	138.9197	34.16%	-	0.9588	
TSO^66^	62.86	70.21%	0.8600	0.9704	
HPO^62^	75.52	64.21%	0.8900	0.97	
HGWOPSO^67^	85.5	59.47%	0.879	0.967	

Table 9. summarizes the minimum SI, voltage absolute, and losses reduction for several studies against the presented research, which proves that the introduced approaches represented the best results; also, the partitioning technique is applied rather than the other studies.

**Table 9:** Comparison between the paper results and the other results for IEEE 118-bus PDN.

**Algorithm**	${{\boldsymbol{T}}{\boldsymbol{P}}}_{{\boldsymbol{l}}{\boldsymbol{o}}{\boldsymbol{s}}{\boldsymbol{s}}}$**(kW)**	${{\boldsymbol{T}}{\boldsymbol{P}}}_{{\boldsymbol{l}}{\boldsymbol{o}}{\boldsymbol{s}}{\boldsymbol{s}}}$ **reduction %**	${{\boldsymbol{S}}{\boldsymbol{I}}}_{{\boldsymbol{m}}{\boldsymbol{i}}{\boldsymbol{n}}}\boldsymbol{ }({\boldsymbol{p}}.{\boldsymbol{u}})$	${{\boldsymbol{V}}}_{{\boldsymbol{m}}{\boldsymbol{i}}{\boldsymbol{n}}}\boldsymbol{ }({\boldsymbol{p}}.{\boldsymbol{u}})$	**Partitioning**
Base case	1298.1	-	0.569	0.8688	
SFO	407.3758	68.61%	0.8398	0.9571	
PO	399.3547	69.23%	0.8399	0.9571	
CTLBO^68^	655.767	-	0.8948	0.9772	
heuristic-based approach^58^	859.9	34%	-	0.9292	
HGAPSO^61^	842.9498	35.06%	0.9610	0.851	
HPO^62^	685.65	47.18%	0.945	0.844	

### Results of converting the benchmark system into a real-time system

#### Daily load profile

**Fig. 14:**
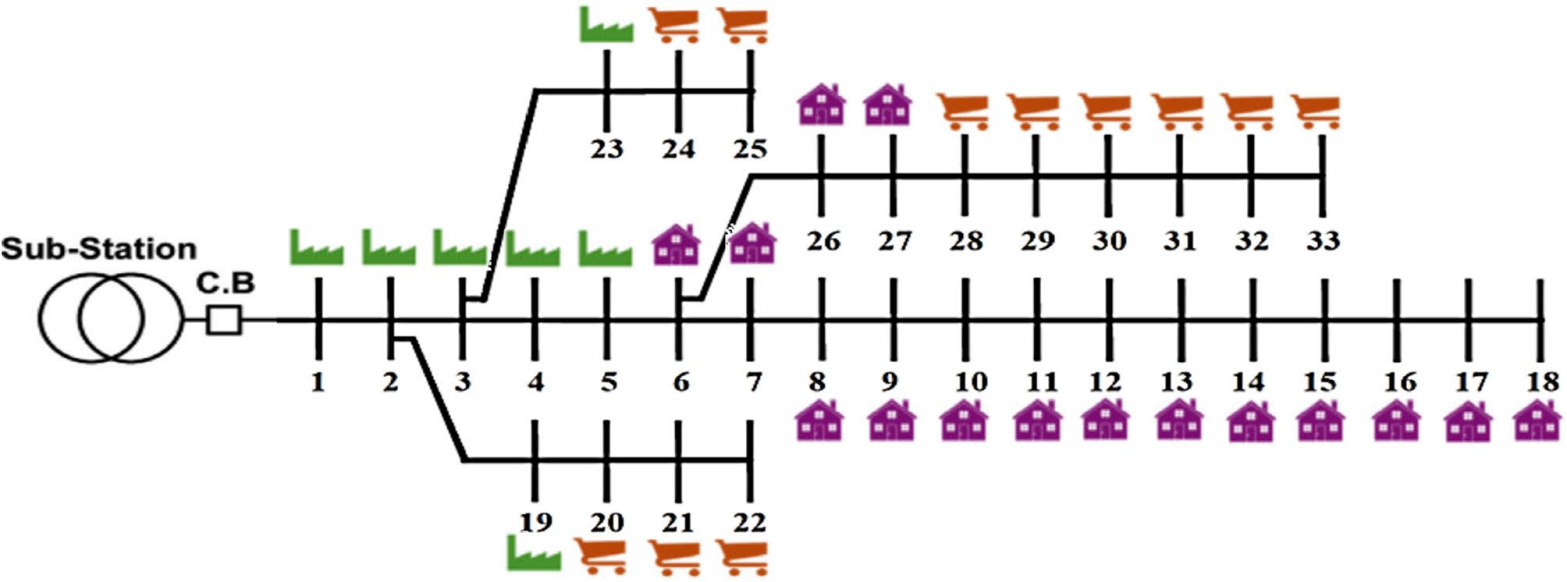
IEEE 33-bus PDN in real-time.

**Fig. 15:**
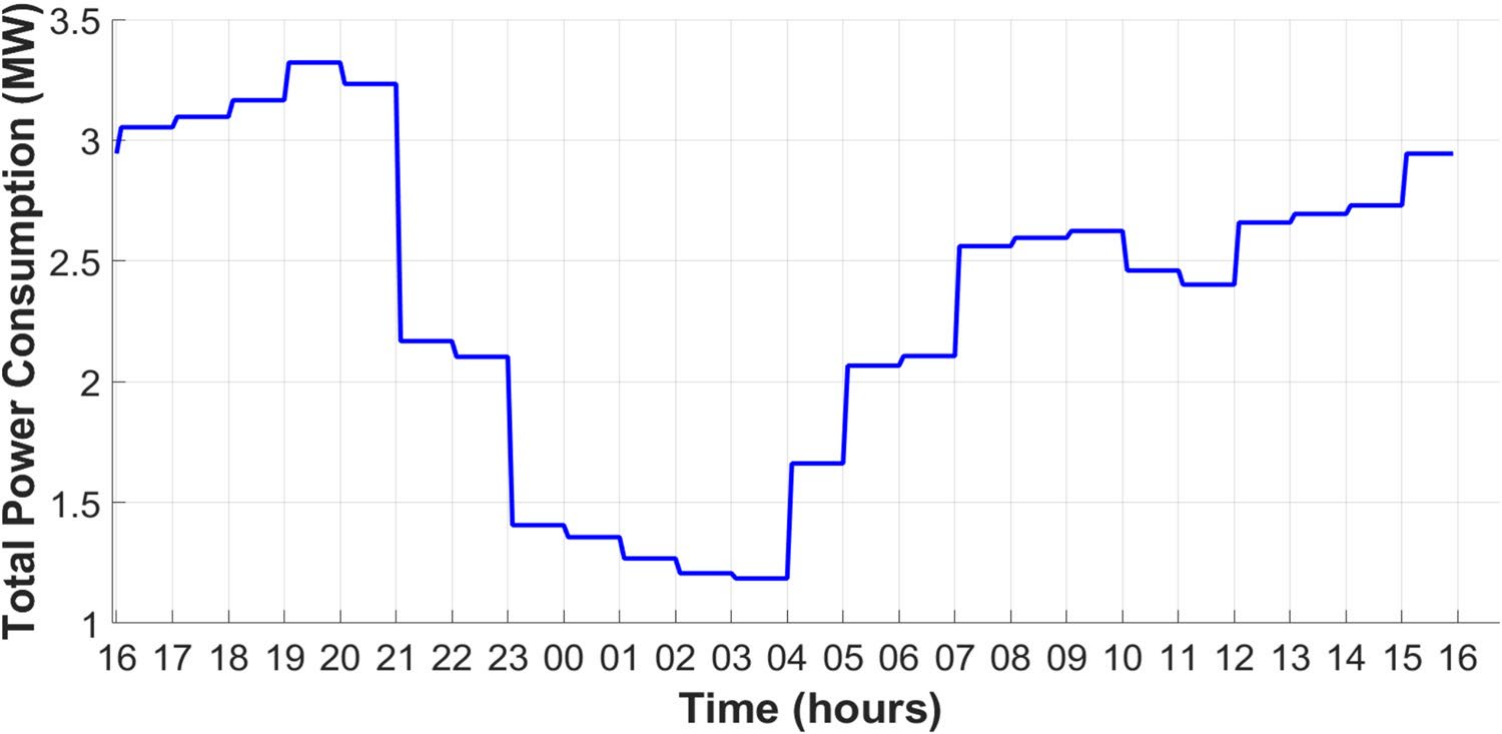
Daily load curve of 33-bus PDN.

The proposed model is divided into VMs, each VM includes a type of load as shown in Figure 14. Hence, the conversion from a static-load system into a real-time system needs a load profile that depends on the type of load in each bus, where this load varies for 24 hours, as represented in Figure 15. From the daily load curve, the maximum demand power reached 3.35 MW at 7 pm for the 33-bus PDN.

#### Electric vehicles data

The applied approach is obtained at the partitioning distribution networks, in which these networks are supplied through a distribution transformer of 23/0.415 kV and 100 kVA. The penetration levels are 100%, 75%, and 50%, with 38 EVs in each CS for each VM that can be linked or not, according to the user’s needs. According to^69^, the EV total number is 264; there are times of arrival and departure, also an initial charging status (ICS), and a required charging status (RCS). All these data are extracted from^54^. Additionally, each EV has a specifies type of battery and charger, such as the capacity of battery: 6, 19.2, and 16 kWh, and charger power: 3.3, 6.6, and 7.2 kW, and the type of charger is an AC charger type 2 (Slow charging).

#### Uncoordinated charging process

**Fig. 16:**
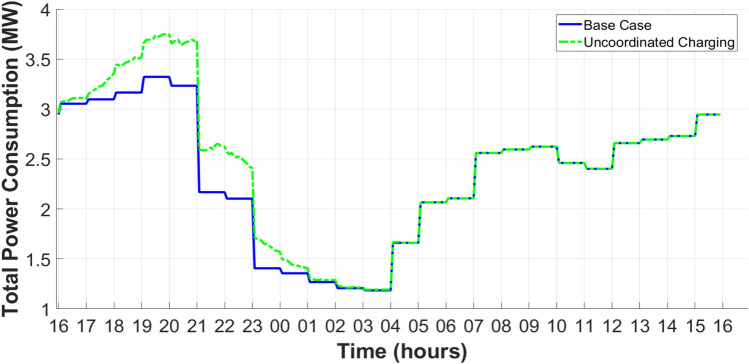
Total power demand considering the uncoordinated EV charging process.

The uncoordinated charging procedure results in a boost in power demand, energy losses, and a decline in absolute voltage. Due to the stochastic charging process of EVs in the PDN, the peak power demand increased from 3.3 MW to 3.75 MW, as presented in Figure 16.

#### Adding wind energy resources in each Virtual microgrid

**Fig. 17:**
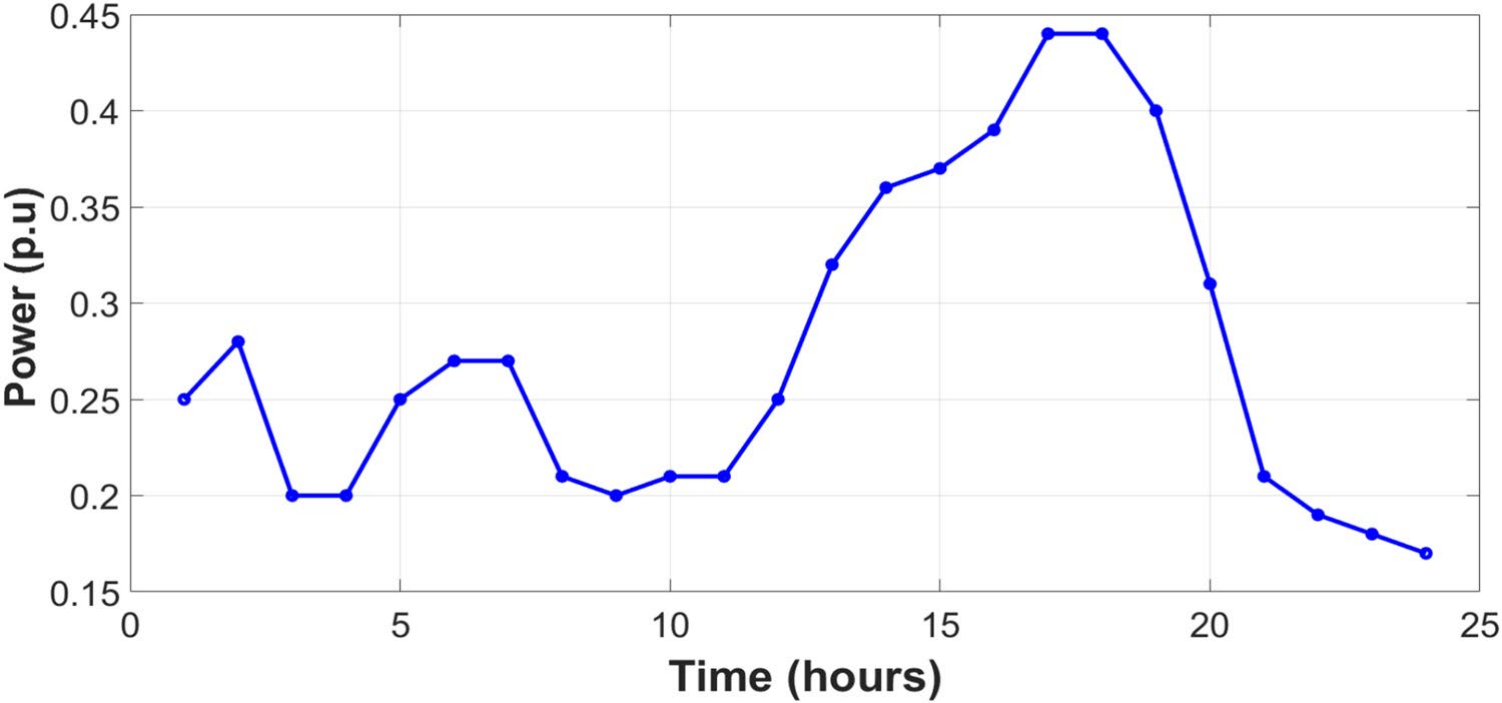
Output power from wind-based DG^70^.

**Fig. 18:**
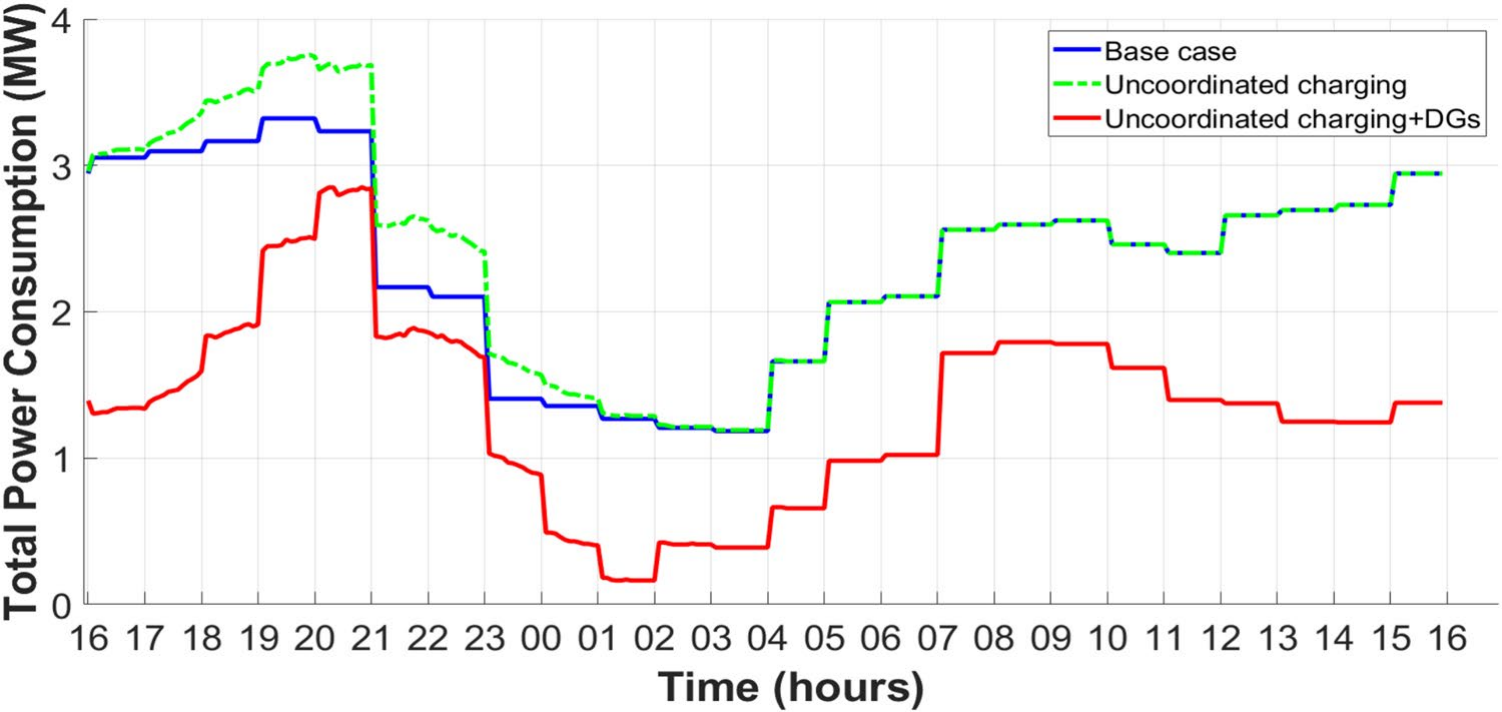
Total power demand after adding DGs for the IEEE 33-bus PDN.

The integration of a wind turbine to a synchronous generator as type-3 of DG types^11^ is an effective method to support system efficiency and reliability to the IEEE 33-bus PDN, where the output power from this DG is shown in the Figure 17. Therefore, these DGs will be added as illustrated in the Figure 18 to prove the reduction of peak power demand, peak power loss, and enhancement in busbar voltages in the highest demand slot. The peak demand and peak loss have decreased from 3.75 MW to 2.87 MW.

## Conclusion

This study introduces a sophisticated optimization framework for the optimal allocation of EV-CSs and resources within PDNs. The proposed methodology integrates the Modified NFA, based on the ECS matrix, to partition the network into electrically cohesive VMs. Each VM is then assigned an EV-CS and a DG to enhance system performance, optimally allocated using the SFO and PO approaches, considering a lowering in total losses and the boosting VSI while maintaining the absolute voltage within its allowable limits, $\pm$ 5%. The outcomes of the 33-bus PDN and 118-bus PDN verify the efficiency of the discussed strategy. Furthermore, in this research paper, the model is converted into a real-time model by integrating the stochastic charging process of EV and enhanced by adding DGs with wind turbines, considering the uncertainties of wind energy. For the 33-bus PDN, the losses are decreased by approximately 81%, with the bottom absolute voltage increased to 0.979 p.u and SI enhanced to 0.92 p.u using the PO technique. Beyond technical development, this work also provides actionable insights for PDN planning. By limiting the penetration of DG to 60% of the total power generated and optimally placing EV-CSs, the proposed approach offers utilities a methodology for integrating DGs without compromising PDN stability. Therefore, it can inform policy and regulatory guidelines related to DG hosting capacity, EV charging infrastructure planning, and renewable integration. Additionally, the 118-bus PDN results show a power loss reduction between 68% and 69%, accompanied by improvements in voltage profile and stability using PO by 0.9571 p.u and SFO by 0.8399 p.u. Among the tested algorithms, PO demonstrates the fastest convergence speed and lowest execution time, outperforming both SFOA and the conventional PSO. In addition, the algorithm’s results are subjected to the Wilcoxon statistical test to ensure the robustness of the proposed techniques. These findings underscore the robustness and scalability of the suggested method. This work has several limitations that should be acknowledged. First, the optimization objectives were limited to power loss diminution and voltage profile enhancement, excluding economic and environmental aspects. In addition, the scalability of the introduced approach to very wide-scale networks remains to be explored. Finally, validation was restricted to simulation results, and further field-based testing is required to confirm practical applicability. Future extensions of this study will incorporate multi-objective cost-based optimization and assess the role of bidirectional energy flows, including Vehicle-to-Grid (V2G) and Grid-to-EV (G2EV) interactions. Moreover, the proposed framework will be applied to a real-world Egyptian PDN to assess its practical deployment potential.

**F**uture work

Although the presented allocation strategy optimized by optimization approaches (SFO, PO, and PSO) has evidence a promising result in improving the PDN performance, various future work directions can be followed: (i) Allocate the EV-CS as a bidirectional power flow facility (charging/discharging) using a novel metaheuristic optimization algorithm (ii) Hybrid metaheuristic approaches (PO with SFO or with PSO) to coordinate charging/discharging after EV-CSs and DGs allocation (iii) Optimal scheduling charging/discharging after EV-CSs siting then allocate different types of FACT and fuel cell to support PDN performance (iv) Novel clustering method to enhance system efficiency using K-means algorithm to get the optimal partitioning network and detect which zone is the best to allocate EV-CS, DG and fuel cell.

## Data Availability

The authors would like to confirm that all data generated or analysed during this study are included within the entire text of the presented paper.

## List of symbols

$${Z}_{ij}^{E}$$: Effective impedance $$i and j$$
${Z}_{ij}$: Element of z-bus matrix between bus $i and j$
${N}_{B}$: Total buses number
${N}_{L}$: Total lines number
${C}_{ij}$: Power capacity of line between bus $i\: and\: j$
${P}_{\mathit{max}l}$: Maximum power of line $l$
${PTDF}_{ij}^{l}$: Power transmitted distribution factor of line $l$ injected to bus $i$ withdrawn from bus $j$
$$\alpha and \beta$$: Proportion coefficients
${ECS}_{ij}$: Electrical coupling strength between bus $i and j$
${Y}_{ij}$: Equivalent admittance between $i and j$
$\overline{\mathrm{C} }$: Average power capacity
$\overline{\mathrm{Y} }$: Average equivalent admittance
${Q}_{e}$: Total electrical modularity
$m$: Total ECS of the network
$$\delta$$: Kronecker delta
${c}_{i}$: Number of community of bus $i$
${\Delta Q}_{e}$: Change in electrical modularity
${I}_{l, Thermal}$: Thermal limit of line $l$
${R}_{l}$: Line resistance of line $l$
${P}_{j}$: Real power of bus $j$
${Q}_{j}$: Imaginary power of bus $j$
${X}_{ij}$: Reactance between $i$ and $j$
${V}_{i}$: Absolute voltage of bus $i$
${P}_{Gen,a}$: Active power generation by $a$ generator in the system
${Q}_{Gen,a}$: Reactive power generation by $a$ generator in the system
$T{P}_{loss}$: Total real power losses
$T{Q}_{loss}$: Total imaginary power losses
${Q}_{Load,c}$: Reactive power of load $c$
${P}_{Load,c}$: Real power of load $c$
$F$: Multi-objective function
${f}_{1}$: Minimum value of total active power losses
${f}_{2}$: Maximum value of the voltage stability index
${w}_{1}$ and ${w}_{2}$: Weight factors, thus $({w}_{1}+{w}_{2}=1)$
${SI}_{j}$: Stability index of bus $j$
${I}_{l}$: Line current for line $l$
${I}_{DG}$: Current injected from DG
${I}_{CS}$: Current draw by CS
${LLI}_{max}$: Maximum line loadability index
${VD}_{max}$: Maximum voltage deviation

## Abbreviations

PDN: Power distribution network
DG: Distributed generator
EV-CS: Electric vehicle charging station
NFA: Newman fast algorithm
VM: Virtual microgrid
ECS: Electrical coupling strength
PO: Puma optimization
SFO: Starfish optimization
PSO: Particle swarm optimization
ICS: Initial charge status
RCS: Required charge status
